# Comparison of community- and healthcare-associated methicillin-resistant *Staphylococcus aureus* isolates at a Chinese tertiary hospital, 2012–2017

**DOI:** 10.1038/s41598-018-36206-5

**Published:** 2018-12-17

**Authors:** Haiying Peng, Dengtao Liu, Yuhua Ma, Wei Gao

**Affiliations:** Department of Clinical Laboratory, Linyi People’s Hospital, Linyi, Shandong China

## Abstract

The transmission between community-associated (CA-) and healthcare-associated (HA-) methicillin-resistant *Staphylococcus aureus* (MRSA) has increased the challenge of infection control. To understand the clonal evolution and transmission of MRSA isolates, we compared the characteristics of 175 CA-MRSA and 660 HA-MRSA strains at a Chinese tertiary hospital in 2012–2017. Antibiotic susceptibility was performed on VITEK system, the genetic background of the isolates was characterized by SCC*mec*, *spa*, and MLST typing, while virulence determinants were screened using conventional PCR. Although more than 70% of the CA-MRSA isolates were erythromycin and clindamycin resistant, CA-MRSA was more susceptible than HA-MRSA to most of the antibiotics tested. ST239-MRSA-III-t030 (30%) was the most prevalent clone among HA-MRSA, while ST59-MRSA-IVa-t437 (28.8%) was the major clone among CA-MRSA. Notably, ST59-MRSA-IVa-t437 accounted for 6.7% of the chosen HA-MRSA isolates. Additionally, difference in virulence gene content was found between the CA- and HA-MRSA strains. In conclusion, epidemiological characteristics were largely different between CA- and HA-MRSA. Although ST239-MRSA-III-t030 is still the predominant clone among HA-MRSA, the community clone ST59-MRSA-IVa-t437 has the potential of becoming an essential part of HA-MRSA in the region tested.

## Introduction

*Staphylococcus aureus* (*S*. *aureus*) is one of the most frequent causal pathogens of both community- and hospital-acquired infections^1^. Staphylococcal infections, particularly those caused by methicillin-resistant *S*. *aureus* (MRSA) have increased the morbidity and mortality of patients due to the cumbersome treatment required^2,3^.

Since the first case was reported in 1961 in the United Kingdom^4^, MRSA has been recognized to be most often associated with various infections in patients exposed to nosocomial settings, which is known as healthcare-associated (HA-) MRSA. The advent of community-associated (CA-) MRSA has given rise to a substantial change in the epidemiology of MRSA isolates that has observed during the past decade. The increasing number of infections caused by CA-MRSA in community settings has attracted much attention from scientists worldwide^5^. Although CA-MRSA is often defined clinically through hospital stays and an absence of risk factors for HA-MRSA infections, it has also been distinguished from HA-MRSA through the possession of unique drug resistance patterns and molecular characteristics^6^. Generally, HA-MRSA typically belongs to SCC*mec* I, II and III^7,8^, while CA-MRSA carries SCC*mec* IV or V^8^. Despite the possession of resistance to β-lactam antimicrobial drugs, CA-MRSA traditionally remains resistant to fewer categories of antibiotics than HA-MRSA^9^. In addition, most CA-MRSA isolates carry the *lukSF-PV* (alias *pvl*) genes encoding a bi-component toxin cytolytic toward neutrophils^10,11^, although the correlation between the presence of *lukSF-PV* genes and the clinical outcome remains controversial^11^.

It is a well-established fact that *S*. *aureus* is notorious for its ability to produce a series of virulence factors. However, the foundation of virulence in CA-MRSA is still poorly understood although higher virulence is distinctive of CA-MRSA^12,13^. Li *et al*.^12^ reported that the community clone ST59 is more virulent than hospital associated lineages, and revealed that Agr, α-toxin, and PSMα significantly contributed to the pathogenesis of ST59 CA-MRSA infections. However, it remains to be seen whether other virulence factors produced by *S*. *aureus* contribute to CA-MRSA infections. Therefore, a comparison of virulence gene profiles between HA- and CA-MRSA isolates may help to understand the genetic basis underlying the possible association between some CA-MRSA infections and poor clinical outcome.

A number of studies have described the antimicrobial activities of various agents and the molecular features of CA-MRSA isolates, but few have directly compared CA- and HA-MRSA at the same region. In order to understand the clonal evolution and transmission of MRSA isolates, we compared antimicrobial susceptibilities, molecular characteristics and the occurrence of virulence factors of CA- and HA-MRSA strains at a Chinese tertiary hospital from 2012 to 2017.

## Results

### Clinical characteristics

Of the 835 MRSA isolates detected, 660 (79.1%) and 175 (20.9%) were identified as HA- and CA-MRSA strains, respectively, based on the definitions mentioned in Materials and Methods. Both CA- and HA-MRSA isolates were positive for the *mecA* gene. From clinical medical records, it was found that skin and soft tissue infections (SSTI), respiratory tract and trauma wounds were the top three sites of CA-MRSA infection, accounting for 59.4% (104/175) of all CA-MRSA cases. While 47.1% (311/660) of HA-MRSA isolates were from the respiratory tract. Moreover, 83 (47.4%) CA-MRSA and 351 (53.2%) HA-MRSA strains were obtained from male patients, and the remaining isolates were obtained from female patients.

### Resistance profiles of CA- and HA-MRSA

The resistance patterns of CA- and HA-MRSA isolates to the antibiotics tested are given in Table 1. All MRSA isolates were resistant to the β-lactam antibiotics tested (oxacillin and penicillin). CA-MRSA had high sensitivity to most of the antibiotics including vancomycin (100%), linezolid (100%), tigecycline (100%), quinupristin/dalfopristin (99.4%), ciprofloxacin (94.3%), gentamicin (93.7%), rifampicin (92.6%) and nitrofurantoin (90.9%). On the contrary, the majority of CA-MRSA strains were resistant to clindamycin (88.6%) and erythromycin (78.3%). All HA-MRSA isolates were susceptible to vancomycin, linezolid, tigecycline and quinupristin/dalfopristin. However, only 8.3% of HA-MRSA isolates were susceptible to clindamycin. In addition, the CA-MRSA strains were more susceptible to ciprofloxacin, tetracycline, TMP/SMX (trimethoprim/sulfamethoxazole), rifampicin, gentamicin, nitrofurantoin and levofloxacin than the HA-MRSA strains [p < 0.01 for all comparisons].

**Table 1:** Antimicrobial susceptibilities of HA-MRSA and CA-MRSA isolates.

Antibiotic	CA-MRSA (n = 175)			HA-MRSA (n = 660)			*P* value^e^
	R^b^, n (%)	I^c^, n (%)	S^d^, n (%)	R^b^, n (%)	I^c^, n (%)	S^d^, n (%)	
Penicillin	175 (100.0)	0 (0)	0 (0)	660 (100.0)	0 (0)	0 (0)	—
Oxacillin	175 (100.0)	0 (0)	0 (0)	660 (100.0)	0 (0)	0 (0)	—
Erythromycin	137 (78.3)	8 (4.6)	30 (17.1)	542 (82.1)	16 (2.4)	102 (15.5)	>0.05
Ciprofloxacin	10 (5.7)	0 (0)	165 (94.3)	568 (86.1)	0 (0)	92 (13.9)	<0.01
Tetracycline	27 (15.4)	2 (1.1)	146 (82.4)	541 (82.0)	3 (0.5)	116 (17.6)	<0.01
Clindamycin	155 (88.6)	0 (0)	20 (11.4)	591 (89.5)	14 (2.1)	55 (8.3)	>0.05
TMP/SMX^a^	18 (10.3)	0 (0)	157 (89.7)	401 (60.8)	0 (0)	259 (39.2)	<0.01
Rifampicin	6 (3.4)	7 (4.0)	162 (92.6)	198 (30.0)	9 (1.4)	453 (68.6)	<0.01
Gentamicin	11 (6.3)	0 (0)	164 (93.7)	433 (65.6)	0 (0)	227 (34.4)	<0.01
Nitrofurantoin	10 (5.7)	6 (3.4)	159 (90.9)	119 (18.0)	2 (0.3)	539 (81.7)	<0.01
Vancomycin	0 (0)	0 (0)	175 (100.0)	0 (0)	0 (0)	660 (100.0)	—
Levofloxacin	51 (29.1)	2 (1.1)	122 (69.7)	384 (58.2)	0 (0)	276 (41.8)	<0.01
Linezolid	0 (0)	0 (0)	175(100.0)	0 (0)	0 (0)	660 (100.0)	—
Tigecycline	0 (0)	0 (0)	175 (100.0)	0 (0)	0 (0)	660 (100.0)	—
Quinupristin/Dalfopristin	0 (0)	1 (0.6)	174 (99.4)	0 (0)	0 (0)	660 (100.0)	—

^a^TMP-SMX, trimethoprim/sulfamethoxazole. ^b^R, Resistant. ^c^I, Intermediate. ^d^S, Susceptible. ^e^The resistance rates of antibiotics among CA-MRSA compared with those among HA-MRSA.

### Molecular characterizations of CA- and HA-MRSA

SCC*mec* IVa was the predominant epidemic type among CA-MRSA, accounting for 70.9% (124/175). Other SCC*mec* types identified among the CA-MRSA isolates included type V (15.4%, 27/175) and type III (10.3%, 18/175). In addition, 6 (3.4%) CA-MRSA isolates were not identifiable through the SCC*mec* typing method used. In contrast to CA-MRSA genotypes, a majority of HA-MRSA isolates carried SCC*mec* III (58.3%, 385/660), followed by type II (25.5%, 168/660), type IVa (11.7%, 77/660), type I (0.6%, 4/660) and type V (0.6%, 4/660), while twenty-two (3.3%) isolates were untypeable. It should be noted that CA-MRSA strains harboring SCC*mec* III showed more resistance to antibiotics that were similar to those that the HA-MRSA isolates were resistant to, whereas some HA-MRSA isolates carrying SCC*mec* IVa only showed resistance to oxacillin, penicillin and erythromycin.

By sequence analysis of the polymorphic X region of the *spa* gene (see Supplementary File S1), 20 *spa* types were yielded from 175 CA-MRSA isolates. The type t437 was identified in 58.9% (103/175) of CA-MRSA isolates and was the most common *spa* type, followed by t002 (7.4%, 13/175), t030 (5.7%, 10/175), t034 (5.1%, 9/175), t2310 (5.1%, 9/175), t309 (4.6%, 8/175), t441 (2.3%, 4/175) and t12147 (1.7%, 3/175). Each of the remaining *spa* types were represented in less than 3 isolates. Among the 660 HA-MRSA isolates, 22 *spa* types were identified, of which type t030 (44.5%, 294/660), t002 (19.8%, 131/660), t437 (10.6%, 70/660) and t037 (8.5%, 56/660) were the four major *spa* types.

Two hundred randomly chosen CA- and HA-MRSA strains underwent multilocus sequence typing (MLST) and the evolutionary and genetic diversity of these strains are summarized in Tables 2 and 3. A total of 9 different clonal complexes (CCs) within 14 distinct sequence types (STs) were identified for CA-MRSA. ST59 (belonging to CC59) was the predominant type accounting for nearly half (52.5%) of the CA-MRSA isolates. Less than 10 each of the isolates contained the remaining 13 STs (ST338, ST1, ST188, ST5, ST1507, ST149, ST398, ST88, ST239, ST22, ST217, ST121 and ST9) (Table 2). For the 120 HA-MRSA isolates, MLST analysis revealed 12 distinct STs, of which ST239 (61.7%) was the most predominant type. The rest of the STs included ST5 (16.7%), ST59 (10.0%), ST398 (3.3%), ST105 (2.5%), ST2590 (0.8%), ST1777 (0.8%), ST30 (0.8%), ST88 (0.8%), ST1 (0.8%), ST45 (0.8%) and ST7 (0.8%) (Table 3). ST1, ST239, ST398, ST5, ST59 and ST88 were observed in both CA- and HA-MRSA strains, whereas ST121, ST1507, ST188, ST217, ST22, ST338, ST72 and ST9 were only present among the CA-MRSA strains, and ST105, ST1777, ST217, ST2590, ST30, ST45 and ST7 were found only among the HA-MRSA strains.

**Table 2:** Molecular characteristics of chosen CA-MRSA isolates described in this study (n = 80).

No. of isolates	Clonal complex (CC)	ST (n, %)	*spa* type (n)	SCC*mec* type (n)
42	CC59	ST59 (37, 52.5)	t437 (34), t441 (2), t034 (1)	IVa (28), V (5), III (4)
		ST338 (5, 6.25)	t437 (4), t441(1)	IVa (4), III (1)
10	CC1	ST1 (6, 7.5)	t127(6)	IVa (4), III (2)
		ST188 (4, 5.0)	t2310 (2), t189 (1), t12147 (1)	IVa (4)
7	CC5	ST5 (3, 3.75)	t002 (3)	IVa (2), N (1)
		ST1507(2, 2.5)	t664 (2)	V (2)
		ST149 (2, 2.5)	t437 (2)	III (1), IVa (1)
6	CC398	ST398 (6, 7.5)	t034 (5), t011 (1)	IVa (6)
5	CC88	ST88 (5, 6.25)	t2310 (2), t7637 (2), t15796 (1)	IVa (3), V (1), N (1)
5	CC239	ST239 (5, 6.25)	t030 (5)	III (4), IVa (1)
3	CC22	ST22 (2, 2.5)	t5983 (2)	IVa (2)
		ST217 (1, 1.25)	t309 (1)	III (1)
1	CC121	ST121 (1, 1.25)	t159 (1)	IVa (1)
1	CC9	ST9 (1, 1.25)	t309 (1)	V (1)

**Table 3:** Molecular characteristics of HA-MRSA isolates described in this study (n = 120).

No. of isolates	Clonal complex (CC)	ST (n, %)	*spa* type (n)	SCC*mec*type (n)
74	CC239	ST239 (74, 61.7)	t030 (56), t037(14), t632(3), t7576 (1)	III (56), II (18)
24	CC5	ST5 (20, 16.7)	t002 (14), t570 (3), t187 (2), t2460 (1)	II (18), III (1), N (1)
		ST2590 (1, 0.8)	t002 (1)	II (1)
		ST105 (3, 2.5)	t688 (3)	II (3)
12	CC59	ST59 (12, 10.0)	t437 (11), t1451 (1)	IVa (8), III (4)
4	CC398	ST398 (4, 3.3)	t034 (4)	III (2), II (2)
2	CC30	ST1777 (1, 0.8)	t3651 (1)	II (1)
		ST30 (1, 0.8)	t019 (1)	IVa (1)
1	CC88	ST88 (1, 0.8)	t7637 (1)	I (1)
1	CC1	ST1 (1, 0.8)	t114 (1)	III (1)
1	CC45	ST45 (1, 0.8)	t2637 (1)	IVa (1)
1	CC7	ST7 (1, 0.8)	t091 (1)	IVa (1)

Certain association was observed among specific SCC*mec*, ST and *spa* types in both CA- and HA-MRSA. For example, the genotypes of ST239-MRSA-III-t030, ST239-MRSA-III-t037 and ST5-MRSA-II-t002 accounted for 30.0%, 11.7% and 10.0%, respectively, of the chosen HA-MRSA strains, whereas ST59-MRSA-IVa-t437 accounted for 28.8% and 6.7% of the chosen CA- and HA-MRSA strains respectively. The virulence gene distribution and antimicrobial resistance profiles of these five specific molecular types of MRSA isolates are listed in Tables 4 and 5 respectively. In particular, the antimicrobial resistance profiles of ST239-MRSA-III-t030 (HA), ST5-MRSA-II-t002 (HA) and ST239-MRSA-III-t037 (HA) clone conformed to those of HA-MRSA analyzed above, with the exclusion that ST5-MRSA-II-t002 clones were more susceptible to nitrofurantoin. Of note, ST59-MRSA-IVa-t437 clones collected from nosocomial settings possessed most of characteristics of antimicrobial resistance representative of CA-MRSA. In comparison, ST59-MRSA-IVa-t437 (HA) isolates were more likely to be resistant to TMP/SMX and gentamicin, while ST59-MRSA-IVa-t437 (CA) were more likely to be resistant to clindamycin and levofloxacin. As for carriage of virulence factors, ST5-MRSA-II-t002 (HA) clones were more likely to possess the superantigen gene (e.g., *tst*, *sec*, *seg*, *seh* and *sei*) compared to other HA clones. The detection rates of the *sea*, *seg*, *seq* and *etb* genes also showed difference between ST59-MRSA-IVa-t437 clones of CA- and HA-MRSA.

**Table 4:** Virulence gene distribution among the five specific molecular types of MRSA isolates.

	Isolates (n)	*icaA* (%)	*icaD* (%)	*clfA* (%)	*clfB* (%)	*fnbA* (%)	*fnbB* (%)	hla (%)	*hlb* (%)	*tst* (%)	*sea* (%)	*seb* (%)	*sec* (%)	*sed* (%)	*see* (%)	*seg* (%)	*seh* (%)	*sei* (%)	*sek* (%)	*seq* (%)	*lukSF-PV* (%)	*PSMα* (%)	*eta* (%)	*etb* (%)
ST239-MRSA-III-t030 (HA-MRSA)	36	100	100	100	94.4	55.6	77.8	100	66.7	0	33.3	0	0	0	0	0	0	5.6	0	5.6	0	91.7	0	0
ST5-MRSA-II-t002 (HA-MRSA)	12	100	100	100	100	83.3	100	100	41.7	58.3	25	0	33.3	0	0	91.7	8.3	75	41.7	0	0	75	0	0
ST239-MRSA-III-t037 (HA-MRSA)	14	100	100	100	100	50	100	100	50	0	28.6	0	0	0	0	0	0	0	0	50	0	100	0	0
ST59-MRSA-IVa-t437 (HA-MRSA)	8	100	100	100	100	100	100	100	100	0	12.5	87.5	0	0	0	0	0	12.5	100	0	100	100	0	0
ST59-MRSA-IVa-t437 (CA-MRSA)	23	100	100	100	100	100	100	100	87	0	0	95.7	0	0	0	8.7	4.3	0	91.3	78.3	82.6	100	0	21.7

**Table 5:** Antimicrobial resistance profiles among the five specific molecular types of MRSA isolates.

	ST239-MRSA-III-t030 (HA, n = 36)	ST5-MRSA-II-t002 (HA, n = 12)	ST239-MRSA-III-t037 (HA, n = 14)	ST59-MRSA-IVa-t437 (HA, n = 8)	ST59-MRSA-IVa-t437 (CA, n = 23)
Penicillin (%)	100	100	100	100	100
Oxacillin (%)	100	100	100	100	100
Erythromycin (%)	72.2	58.3	78.6	100	82.6
Ciprofloxacin (%)	94.4	41.7	78.6	0	0
Tetracycline (%)	94.4	58.3	71.4	12.5	13.0
Clindamycin (%)	91.7	91.7	64.3	25	78.3
TMP/SMX (%)	41.7	33.3	64.3	25	0
Rifampicin (%)	22.2	16.7	7.1	0	0
Gentamicin (%)	94.4	66.7	50	25	0
Nitrofurantoin (%)	8.3	0	21.4	0	0
Vancomycin (%)	0	0	0	0	0
Levofloxacin (%)	52.8	41.7	85.7	0.0	21.7
Linezolid (%)	0	0	0	0	0
Tigecycline (%)	0	0	0	0	0
Quinupristin/ Dalfopristin (%)	0	0	0	0	0

### Screening for virulence factors

Both CA- and HA-MRSA isolates were subjected to detection of virulence genes. All virulence genes except *sed*, *see*, *seh*, and *eta* genes were identified in more than 5 isolates. All isolates harbored no less than 4 detected virulence genes. The *lukSF-PV* genes were present in 53.1% (91/175) of CA-MRSA strains, all of which carried SCC*mec* IVa or V, except for one isolate. In contrast to CA-MRSA, a majority (582/660, 88.2%) of HA-MRSA strains were *lukSF-PV*-negative. The *tst* gene was present in 26.4% of HA-MRSA isolates, whereas only one CA-MRSA isolate was positive for the *tst* gene (Table 6). In addition, the *fnbA*, *fnbB*, *seb*, *sei*, *sek*, and *seq* genes presented more commonly in CA-MRSA than those in HA-MRSA. While *clfA*, *clfB* and *sec* genes were more frequently found among the HA-MRSA isolates (Table 6). All CA- and HA-MRSA isolates, with the exception of five and four isolates, respectively, were positive for *hla*. Adhesion genes were identified in most of the CA- and HA-MRSA isolates, although the two types of MRSA had different detection rates of *clfA*, *clfB*, *fnbA*, *fnbB* genes (Table 6).

**Table 6:** Distribution of toxin genes in CA- and HA-MRSA strains.

Virulence factor	CA-MRSA (n = 175)	HA-MRSA (n = 660)	*P* value
	n (%)	n (%)	
**Adhesion gene**			
*icaA*	162 (92.6)	619 (93.8)	>0.05
*icaD*	162 (92.6)	626 (94.8)	>0.05
*clfA*	138 (78.9)	601(91.1)	<0.01
*clfB*	127 (72.6)	655 (99.2)	<0.01
*fnbA*	143 (81.7)	361 (54.7)	<0.01
*fnbB*	142 (81.1)	479 (72.6)	<0.05
**Hemolysin gene**			
*hla*	171 (97.7)	655 (99.2)	>0.05
*hlb*	114 (65.1)	467 (70.8)	>0.05
**Superantigen gene**			
*tst*	1 (0.6)	174 (26.4)	<0.01
*sea*	13 (7.4)	51 (7.7)	>0.05
*seb*	84 (48.0)	57 (8.6)	<0.01
*sec*	5 (2.9)	60 (9.1)	<0.01
*sed*	0	0	—
*see*	0	0	—
*seg*	5 (2.9)	29 (4.4)	>0.05
*seh*	1 (0.6)	1 (0.2)	>0.05
*sei*	24 (13.7)	54 (8.2)	<0.05
*sek*	39 (22.3)	32 (4.8)	<0.01
*seq*	33 (18.9)	9 (1.4)	<0.01
**Other virulence gene**			
*lukSF-PV*	93 (53.1)	78 (11.8)	<0.01
*PSMα*	123 (70.3)	482 (73.0)	>0.05
*eta*	0	0	—
*etb*	7 (4.0)	17 (2.6)	>0.05

*icaA*: intercellular adhesion gene A, *icaD*: intercellular adhesion gene D, *clfA:* clumping factor A gene, *clfB*: clumping factor A gene, *fnbA*: fibronectin-binding protein A gene, *fnbB*: fibronectin-binding protein B gene, *hla*: alpha- haemolysin gene, *hld*: delta-haemolysin gene, *tst*: toxic shock syndrome toxin -1 gene, *sea*~*see*, *seg*~*sei*, *sek*, *seq*: the genes of staphylococcal enterotoxins (SEs), *lukSF-PV*: genes encoding Panton-Valentine Leukocidin S and F subunit, *PSMα*: phenol-soluble modulin α gene, *eta*: exfoliative toxin A gene, *etb*: exfoliative toxin B gene.

## Discussion

CA-MRSA, an organism that has been emerging worldwide, is notorious for its disease-causing potential including diseases such as SSTIs and necrotizing pneumonia. Recent reports of CA-MRSA infections with severe presentation and poor outcome among young age groups have been noted^5^. Considering the serious clinical consequences of MRSA infection and difference in suitable antimicrobial treatments for CA- and HA-MRSA^14^, it is necessary to distinguish between these two types of *S*. *aureus*, and to understand their molecular characteristics, antimicrobial susceptibility patterns and virulence gene profiles. This will help in taking effective measures to prevent infection and reduce transmission in the community and at healthcare facilities.

*In vitro* determination of antibiotic susceptibility patterns of MRSA is essential for rational selection of antibiotics used in treatment of staphylococcal infections. Our results revealed that CA-MRSA strains were more susceptible to ciprofloxacin, tetracycline, TMP/SMX, rifampicin, gentamicin, nitrofurantoin and levofloxacin, which is consistent with previous studies^9,15^. SSTIs occur frequently and were the leading source of infection among the CA-MRSA isolates in this study. Most SSTIs can be treated using topical antibiotics but a small proportion of patients will still need systematic treatment. The European guidelines recommend that vancomycin, teicoplanin, linezolid, daptomycin, tigecycline, and ceftaroline be used in the treatment of SSTIs^16^. According to the antibiotic susceptibility tests of this study, both CA- and HA-MRSA isolates continued to exhibit excellent susceptibility to vancomycin, linezolid and tigecycline. This is in accordance with previous study^15^ and can also serve as empirical therapeutic evidence for the reliability of these antibiotics for SSTIs. Additionally, our results suggest that penicillin, oxacillin, erythromycin and clindamycin are no longer wise choices for SSTI treatment. Together with the results of a recent study in China, where it was shown that ST59 CA-MRSA strains were resistant to erythromycin, clindamycin and penicillin^9^, our findings indicate that antibiotic abuse in the community of China may have resulted in selective pressure for these agents.

CA-MRSA is recovered from people who have not been hospitalized or had a medical procedure during the past year^17^. Previous studies have shown that ST59-MRSA-IV was a widespread primary CA-MRSA clone in China, which always harbored t437^9,18^. While ST239-MRSA-III (commonly with t030 and t037) and ST5-MRSA-II (commonly with t002) clones comprised most of the HA-MRSA strains found in China^19,20^. Our results are partially consistent with this earlier data, demonstrating that ST239-MRSA-III-t030/t037, ST59-MRSA-IVa-t437 and ST5-MRSA-II-t002 were the predominant clones among HA-MRSA, while ST59-MRSA-IVa-t437 accounted for a larger proportion of CA-MRSA.

A previous study^20^ of MRSA strains from nine hospitals in Shenzhen city revealed that 28.9% of HA-MRSA strains were CC59-MRSA-IV/V-t437 clones that exhibited features of traditional CA-MRSA in China. Similarly, we identified 8 HA-MRSA isolates that all harbored typical CA-MRSA attributes - ST59 genotype, SCC*mec* IVa-bearing and *lukSF-PV* positive. Moreover, four strains isolated from patients who had exposure to the community environment also possessed the characteristics representative of HA-MRSA. This paradoxical finding indicates that ST59 has a general tendency of becoming a common HA-MRSA clone in parallel with ST239, as illustrated by previous reports to China^20–22^. This also suggests that hospital-community transmission of MRSA strains through medical staff and community residents may happen frequently^23^. This underlines the need for surveillance of the alteration of MRSA epidemiology and a need of taking corresponding measures to control staphylococcal infection and transmission.

A study at a tertiary hospital in China (the First Affiliated Hospital, College of Medicine, Zhejiang University) revealed that ST5-MRSA-II-t311 was the most prevalent clone among the strains studied, followed by ST5-MRSA-II-t002 and ST59-MRSA-IV-t437^19^. In addition, a study from Taiwan showed that ST59-MRSA-V-t437 and ST45-MRSA-V-t1081 occupied a large portion of MRSA isolates collected^24^. These studies together with our findings indicate that differences exist in MRSA epidemic situations among hospitals in China.

The pathogenicity of *S*. *aureus* largely relies on the ability to produce a range of virulence factors, including cytolytic toxins, hemolysins, exoenzymes and superantigens^25^. A better knowledge of toxin gene carriage is necessary to explore the virulent basis of MRSA and to institute an effective therapeutic strategy. In this study, differences in virulence gene content was found between CA- and HA-MRSA strains. For instance, of the HA-MRSA isolates, 26.4% possessed the *tst* gene while only 11.8% were *lukSF-PV*-positive. In contrast, only one CA-MRSA isolate harbored the *tst* gene, while 53.1% carried the *lukSF-PV* genes (Table 6). The correlation between the presence of virulence genes and specific molecular types was revealed as well, including the linkage between *lukSF-PV* genes and SCC*mec* IVa or V in CA-MRSA. However, association was not always found between molecular characteristics and virulence genes. Adhesion genes (*icaA*, *icaD*, *clfA*, *clfB*, *fnbA* and *fnbB*) and hemolysin genes (*hla*, *hlb*) were detected in most of the tested isolates, suggesting that these common toxin genes carried by MRSA within various lineages play significant roles in staphylococcal pathogenicity^9^.

Previous studies have reported that the *tst* gene, encoding the toxic shock syndrome toxin-1 (TSST-1), was mostly identified in CC398, CC15, CC188, CC59 and CC8 but rarely detected in CC5 isolates in China^25,26^. However, in a recent study from Suzhou in China, the *tst* gene was identified in 18.0% of all isolates tested, and all but one belonged to CC5^27^. In this study, the *tst* gene was present in 26.4% of HA-MRSA isolates, all of which belonged to CC5. Together with findings from the previous study in which CC5 HA-MRSA isolates had strong association with the *tst* gene^27^, our results further emphasize that CC5 may be an emerging *tst*-harboring clone in China. Further studies of more geographically diverse MRSA strains are warranted to confirm this finding.

Generally, CA- and HA-MRSA are separated from one another based on whether it is collected from a nosocomial setting or a community setting. The specific molecular characteristics and antimicrobial resistance patterns of CA- and HA-MRSA isolates have helped us to identify each of them. However, with the migration of CA-MRSA isolates into healthcare settings^20^, it is increasingly difficult to discriminate between CA- and HA-MRSA and the above definition and method of identification no longer seem accurate. Therefore, a better monitoring method is required to differentiate between traditional HA-MRSA and HA-MRSA derived from community settings.

In summary, this study provides information on the molecular characteristics, antimicrobial susceptibility patterns and virulence gene profiles of CA- and HA- MRSA isolates at a tertiary hospital in China. The definition of CA- and HA-MRSA by genotype may be not appropriate since ST239 and ST59, which are recognized as the major clones of CA- and HA-MRSA strains respectively, are circulating simultaneously in both community and healthcare settings in China. Continuous monitoring of CA- and HA-MRSA is necessary to understand the evolution and transmission of this ubiquitous pathogen.

## Materials and Methods

### Collection of isolates

A total of 835 MRSA isolates including 175 CA-MRSA and 660 HA-MRSA strains were collected from Linyi People’s Hospital, a tertiary hospital in China, between August 2012 and August 2017. These MRSA strains were isolated from clinical sources such as blood, the respiratory tract (sputum, bronchial alveolar lavage fluid, and pharynx swabs), skin and soft tissue (cutaneous abscess and wound secretion), vaginal discharge, stools and urine. The data was analyzed anonymously. All isolates were identified using a VITEK System (BioMérieux, Marcy l′ Etoile, France). MRSA isolates were recognized by their resistance to cefoxitin and confirmed by the presence of *mecA* or *mecC* genes. Subsequently, the MRSA isolates were categorized as CA- or HA-MRSA according to the definition previously proposed^28^. Briefly, CA-MRSA was any MRSA strain that was isolated from an outpatient or an inpatient within 48 hours of admission to hospital, where the host had no medical history of MRSA infection or colonization, dialysis, surgery or insertion of indwelling devices or other risk factors of HA-MRSA infections for the past year. This study was approved by the Ethics Committee of Linyi People’s Hospital and was performed in accordance with relevant local guidelines and regulations. Informed consent was obtained from all participants.

### Antibiotic susceptibility testing

The antibiotic susceptibility testing of all isolates in this study was performed on a VITEK system (BioMérieux, Marcy l′ Etoile, France) according to manufacturer’s instructions. Interpretation of the results was based on Clinical Laboratory Standards Institute (CLSI) antimicrobial susceptibility guidelines^29^. The following antibiotics were tested: penicillin, oxacillin, erythromycin, ciprofloxacin, tetracycline, clindamycin, TMP/SMX, rifampicin, gentamicin, nitrofurantoin, vancomycin, levofloxacin, linezolid, tigecycline and quinupristin/dalfopristin. *S*. *aureus* ATCC25923 was used as a quality control.

### DNA extraction

The pure and distinct colonies of the *S*. *aureus* strains were suspended in 100 µl of TE buffer containing 10 µg of lysostaphin and then vortexed. After incubation at 37 °C for 30 minutes, the mixture was subjected to DNA extraction using the QIAamp DNA mini kit (QIAGEN, Hilden, Germany) according to the manufacturer’s protocol. DNA quantity and purity were tested using a NanoDrop spectrometer (Thermo Fisher Scientific, Waltham, MA, USA).

### Molecular typing

Genetic characterization of all *S*. *aureus* strains was performed by *spa* and SCC*mec* typing, while MLST typing was only preformed on 200 randomly chosen isolates (80 CA-MRSA and 120 HA-MRSA strains) that were representative of each year. The *spa* typing was based on variations in the polymorphic X region of the staphylococcal protein A (*spa*) gene. The X region of each isolate was amplified using PCR with the primers *spa*-1095 F and *spa*-1517 R (see Supplementary Table S2 online)^30^. And then the sequence data of the amplified products were submitted to the website http://www.spaserver.ridom.de to obtain the *spa* type for each isolate^30^. SCC*mec* typing was performed by multiplex PCR assay as described by Zhang *et al*.^31^. PCR conditions were as follows: beginning with a 5-minute denaturation step at 94 °C, followed by 10 cycles of 45 seconds at 94 °C, 45 seconds at 65 °C and 1.5 minutes at 72 °C, and another 25 cycles of 45 seconds at 94 °C, 45 seconds at 55 °C and 1.5 minutes at 72 °C, ending with a final extension step at 72 °C for 10 minutes^31^. For MLST typing, the fragments of seven housekeeping genes (*arcC*, *aroE*, *glpF*, *gmK*, *pta*, *tpi*, and *yqiL*) were sequenced. Sequence types (STs) were determined through the allelic profile assigned by comparing the consensus sequences with data available in the MLST database (http://saureus.mlst.net)^32^. MLST clonal complexes (CCs) were defined using eBURST (http://www.eburst.mlst.net). The primers used for molecular typing are shown in Supplementary Table S2. Amplicon sequencing in both directions was performed at Shanghai Sangon Biotech during the course of *spa* and MLST typing.

### Screening for virulence determinants

All isolates were screened by conventional PCR for the following 23 staphylococcal virulence determinants: adhesion genes (*icaA*, *icaD*, *clfA*, *clfB*, *fnbA*, *fnbB*), hemolysin genes (*hla*, *hlb*), superantigen genes (*tst*, *sea*, *seb*, *sec*, *sed*, *see*, *seg*, *seh*, *sei*, *sek*, *seq*), Panton-Valentine leukocidin genes (*lukSF-PV*), phenol-soluble modulin-α (*PSM*α) and exfoliative toxin genes (*eta*, *etb*) as described previously^33–36^.

### Statistical analysis

All statistical analyses were done using SPSS v.16.0 software (SPSS Inc., USA). The antimicrobial susceptibilities and virulence gene profiles of CA- and HA-MRSA were compared using χ^2^ or Fisher’s exact two-tailed test. A p value < 0.05 was considered statistically significant.

## Electronic supplementary material

Supplementary File S1

Supplementary Table S2
