# Supplementary material for: Comparison of community- and healthcare-associated methicillin-resistant Staphylococcus aureus isolates at a Chinese tertiary hospital, 2012–2017
Source: Sci Rep. 2018 Dec 17;8:17916. doi: 10.1038/s41598-018-36206-5 (PMC6297250; doi:10.1038/s41598-018-36206-5)
Supplement: Supplementary file 1 — Supplementary File S1 [file 41598_2018_36206_MOESM1_ESM.pdf]

Comparison of community- and healthcare-associated methicillin-resistant *Staphylococcus aureus* isolates at a Chinese tertiary hospital, 2012–2017

Haiying Peng [1], Dengtao Liu [1], Yuhua Ma [1] & Wei Gao [1,*]

[1] Department of Clinical Laboratory, Linyi People's Hospital, Linyi, Shandong, China

Haiying Peng: phy1977@126.com

Dengtao Liu: 15969917998@163.com

Yuhua Ma: ma_yuhua@163.com

Wei Gao: gaoweisdly@126.com

***Corresponding author's mailing address:** Department of Clinical Laboratory, Linyi People's Hospital, 27 Jiefang Rd., Linyi, Shandong, People's Republic of China; Zip Code: 276003. Phone: +86-539-8219735. E-mail: gaoweisdly@126.com

Supplementary File S1: Sequencing data of *spa* and MLST typing in this study.

1 Sequencing data of spa typing in this study

>t437
GCACCAAAAGAGGAAGACAATAACAAGCCTGGTAAAGAAGACAACAACAAACCTGG
CAAAGAAGACGGCAACAAGCCTGGTAAAGAAGACAACAACAAACCTGGCAAAGAAG
ACGGCAACAAGCCTGGTAAAGAAGATGGCAACAAACCTGGTAAAGAAGACAACAAA
AAACCTGGTAAAGAAGACGGCAACGGAGTACATGTCGT

>t002
GCACCAAAAGAGGAAGACAACAAAAAACCTGGTAAAGAAGACGGCAACAAACCTGG
CAAAGAAGACGGCAACAAGCCTGGTAAAGAAGACAACAAAAAACCTGGTAAAGAAG
ACGGCAACAAGCCTGGTAAAGAAGACAACAACAAACCTGGCAAAGAAGACGGCAAC
AAGCCTGGTAAAGAAGACAACAACAAGCCTGGTAAAGAAGACGGCAACAAGCCTGG
TAAAGAAGACGGCAACAAACCTGGTAAAGAAGACGGCAACGGAGTACATGTCGT

>t034
GCACCAAAAGAGGAAGACAACAACAAGCCTGGTAAAGAAGACGGCAACAAACCTGG
TAAAGAAGACAACAAAAAACCTGGCAAAGAAGATGGCAACAAACCTGGTAAAGAAG
ACAACAAAAAACCTGGCAAAGAAGATGGCAACAAACCTGGTAAAGAAGACAACAAA
AAACCTGGTAAAGAAGATGGCAACAAGCCTGGTAAAGAAGATGGCAACAAACCTGGT
AAAGAAGACGGCAACGGAATACATGTCGT

> t311
GCACCAAAAGAGGAAGACAACAAAAAACCTGGTAAAGAAGACGGCAACAAACCTGG
CAAAGAAGACGGCAACAAGCCTGGTAAAGAAGACAACAAAAAACCTGGTAAAGAAG
ACAACAACAAACCTGGCAAAGAAGACGGCAACAAGCCTGGTAAAGAAGACAACAAC
AAGCCTGGTAAAGAAGACGGCAACAAGCCTGGTAAAGAAGACGGCAACAAACCTGG
TAAAGAAGACGGCAACGGAGTACATGTCGT

>t114
GCACCAAAAGAGGAAGACAACAACAAACCTGGTAAAGAAGACGGCAACAAACCTGG
TAAAGAAGACAACAAAAAACCTGGTAAAGAAGATGGCAACAAGCCTGGCAAAGAAG
ACAACAACAAACCTGGTAAAGAAGACGGCAACGGAGTACATGTCGT

>t441
GCACCAAAAGAGGAAGACAATAACAAGCCTGGTAAAGAAGACAACAACAAACCTGG
CAAAGAAGACGGCAACAAGCCTGGTAAAGAAGATGGCAACAAACCTGGTAAAGAAG
ACAACAAAAAACCTGGTAAAGAAGACGGCAACGGAGTACATGTCGT

>t7637
GCACCAAAAGAGGAAGACAACAACAAACCTGGTAAAGAAGACAACAACAAGCCTGG
TAAAGAAGACAACAACAAGCCTGGCAAAGAAGACGGCAACAAGCCTGGTAAAGAAG

ACAACAACAAACCTGGTAAAGAAGACAACAACAAACCTGGTAAAGAAGACAACAAC
AAACCTGGTAAAGAAGACAACAAAAAACCTGGTAAAGAAGACAACAAAAAACCTGG
TAAAGAAGACAACAAAAAACCTGGTAAAGAAGACAACAAAAAACCTGGTAAAGAAG
ATGGCAACAAGCCTGGCAAAGAAGACAACAACAAACCTGGTAAAGAAGACGGCAAC
GGAGTACATGTCGT

>t2460
GCACCAAAAGAGGAAGACAACAAAAAACCTGGTAAAGAAGACGGCAACAAGCCTGG
TAAAGAAGACAACAAAAAACCTGGTAAAGAAGACAACAAAAAACCTGGTAAAGAAG
ACGGCAACAAGCCTGGTAAAGAAGACAACAACAAACCTGGCAAAGAAGACGGCAAC
AAGCCTGGTAAAGAAGACGGCAACAAGCCTGGTAAAGAAGACGGCAACAAGCCTGG
TAAAGAAGACGGCAACAAACCTGGTAAAGAAGACGGCAACGGAGTACATGTCGT

>t019
GAGGAAGACAACAACAAGCCTGGTAAAGAAGACGGCAACAAACCTGGTAAAGAAGA
CAACAAAAAACCTGGCAAAGAAGACGGCAACAAACCTGGTAAAGAAGACAACAAAA
AACCTGGCAAAGAAGATGGCAACAAACCTGGTAAAGAAGACGGCAACAAGCCTGGT
AAAGAAGATGGCAACAAGCCTGGT

>t030
GAGGAAGACAACAACAAGCCTGGCAAAGAAGACAACAACAAGCCTGGTAAAGAAGA
CGGCAACAAACCTGGTAAAGAAGACAACAAAAAACCTGGCAAAGAAGATGGCAACA
AGCCTGGTAAAGAAGATGGCAACAAGCCTGGT

>t037
GAGGAAGACAACAACAAGCCTGGCAAAGAAGACAACAACAAGCCTGGTAAAGAAGA
CGGCAACAAACCTGGTAAAGAAGACAACAAAAAACCTGGCAAAGAAGATGGCAACA
AACCTGGTAAAGAAGACGGCAACAAGCCTGGTAAAGAAGATGGCAACAAGCCTGGT

>t091
GAGGAAGACAACAACAAACCTGGTAAAGAAGACGGCAACAAACCTGGCAAAGAAGA
CAACAACAAGCCTGGCAAAGAAGACGGCAACAAGCCTGGTAAAGAAGACAACAAAA
AACCTGGTAAAGAAGACAACAACAAGCCTGGTAAAGAAGACGGCAACAAACCTGGC
AAAGAAGACAACAAAAAACCTGGCAAAGAAGACAACAACAAGCCTGGTAAAGAAGA
CGGCAACAAACCTGGC

>t187
GAGGAAGACAACAAAAAACCTGGTAAAGAAGACGGCAACAAGCCTGGTAAAGAAGA
CAACAAAAAACCTGGTAAAGAAGACGGCAACAAGCCTGGTAAAGAAGACAACAAAA
AACCTGGCAAAGAAGACGGCAACAAGCCTGGTAAAGAAGACAACAACAAGCCTGGT
AAAGAAGACGGCAACAAGCCTGGTAAAGAAGACGGCAACAAACCTGGT

>t2310
GAGGAAGACAACAACAAACCTGGTAAAGAAGACAACAACAAGCCTGGTAAAGAAGA

CAACAACAAGCCTGGCAAAGAAGACGGCAACAAGCCTGGTAAAGAAGACAACAACA
AACCTGGTAAAGAAGACAACAAAAAACCTGGTAAAGAAGACAACAAAAAACCTGGT
AAAGAAGACAACAAAAAACCTGGTAAAGAAGACAACAAAAAACCTGGTAAAGAAGA
TGGCAACAAGCCTGGCAAAGAAGACAACAACAAACCTGGT

>t2637
GAGGAAGACAACAACAAGCCTGGTAAAGAAGACGGCAACAAACCTGGTAAAGAAGA
CAACAAAAAACCTGGCAAAGAAGACGGCAACAAACCTGGTAAAGAAGACAACAACA
AACCTGGTAAAGAAGACGGCAACAAGCCTGGTAAAGAAGACAACAAAAAACCTGGT

>t3167
GAGGAAGACAACAACAAGCCTGGCAAAGAAGACAACAACAAGCCTGGCAAAGAAG
ACAACAACAAGCCTGGTAAAGAAGACGGCAACAAACCTGGTAAAGAAGACAACAAA
AAACCTGGCAAAGAAGATGGCAACAAGCCTGGTAAAGAAGATGGCAACAAGCCTGGT

>t3651
GAGGAAGACAACAACAAGCCTGGCAAAGAAGACAACAACAAGCCTGGTAAAGAAGA
CGGCAACAAACCTGGTAAAGAAGACGGCAACAAACCTGGTAAAGAAGACAACAAAA
AACCTGGCAAAGAAGACGGCAACAAACCTGGTAAAGAAGACAACAAAAAACCTGGC
AAAGAAGATGGCAACAAACCTGGTAAAGAAGACGGCAACAAGCCTGGTAAAGAAGA
CGGCAACAAGCCTGGTAAAGAAGATGGCAACAAGCCTGGT

>t459
GAGGAAGACAACAACAAGCCTGGCAAAGAAGACAACAACAAGCCTGGTAAAGAAGA
CGGCAACAAACCTGGTAAAGAAGACAACAAAAAACCTGGCAAAGAAGATGGCAACA
AGCCTGGT

>t570
GAGGAAGACAACAACAAACCTGGTAAAGAAGACGGCAACAAACCTGGCAAAGAAGA
CGGCAACAAGCCTGGTAAAGAAGACAACAAAAAACCTGGTAAAGAAGACGGCAACA
AGCCTGGTAAAGAAGACAACAACAAACCTGGCAAAGAAGACGGCAACAAGCCTGGT
AAAGAAGACAACAACAAGCCTGGTAAAGAAGACGGCAACAAGCCTGGTAAAGAAGA
CGGCAACAAACCTGGT

>t632
GAGGAAGACAACAACAAGCCTGGTAAAGAAGACGGCAACAAACCTGGTAAAGAAGA
CAACAAAAAACCTGGCAAAGAAGATGGCAACAAGCCTGGTAAAGAAGATGGCAACA
AGCCTGGT

>t688
GAGGAAGACAACAAAAAACCTGGTAAAGAAGACGGCAACAAACCTGGCAAAGAAGA
CGGCAACAAGCCTGGTAAAGAAGACAACAAAAAACCTGGTAAAGAAGACGGCAACA
AGCCTGGTAAAGAAGACGGCAACAAACCTGGT

>t7576
GAGGAAGACAACAACAAGCCTGGCAAAGAAGACAACAACAAGCCTGGTAAAGAAGA
CGGCAACAAACCTGGTAAAGAAGACAACAAAAAACCTGGTAAAGAAGATGGCAACA
AGCCTGGT

>t011
GAGGAAGACAACAACAAGCCTGGTAAAGAAGACGGCAACAAACCTGGTAAAGAAGA
CAACAAAAAACCTGGCAAAGAAGATGGCAACAAACCTGGTAAAGAAGACAACAAAA
AACCTGGTAAAGAAGATGGCAACAAGCCTGGTAAAGAAGATGGCAACAAACCTGGT

>t127
GAGGAAGACAACAACAAACCTGGTAAAGAAGACGGCAACAAACCTGGCAAAGAAGA
CAACAACAAGCCTGGCAAAGAAGACGGCAACAAACCTGGTAAAGAAGACAACAAAA
AACCTGGTAAAGAAGATGGCAACAAGCCTGGCAAAGAAGACAACAACAAACCTGGT

>t1451
GAGGAAGACAACAACAAGCCTGGTAAAGAAGACGGCAACAAACCTGGTAAAGAAGA
CAACAAAAAACCTGGCAAAGAAGATGGCAACAAACCTGGTAAAGAAGACAACAAAA
AACCTGGTAAAGAAGATGGCAACAAACCTGGT

>t159
GAGGAAGACAACAACAAACCTGGCAAAGAAGACAACAAGCCTGGTAAAGAAGACAA
CAACAAACCTGGTAAAGAAGACAACAACAAGCCTGGTAAAGAAGACGGCAACAAGC
CTGGTAAAGAAGACGGCAACAAGCCTGGTAAAGAAGACGGCAACAAACCTGGCAAA
GAAGATGGCAACAAGCCTAGTAAAGAAGACGGCAACAAGCCTGGT

>t189
GAGGAAGACAACAACAAACCTGGTAAAGAAGACGGCAACAAACCTGGCAAAGAAGA
CAACAACAAGCCTGGTAAAGAAGACAACAACAAGCCTGGCAAAGAAGACGGCAACA
AGCCTGGTAAAGAAGACAACAAAAAACCTGGT

>t309
GAGGAAGACAACAAAAAACCTGGTAAAGAAGACGGCAACAAACCTGGCAAAGAAGA
CAACAAAAAGCCTGGCAAAGAAGACGGCAACAAGCCTGGTAAAGAAGATGGCAACA
AACCTGGTAAAGAAGACGGCAACAAGCCTGGTAAAGAAGATGGCAACAAACCTGGTA
AAGAAGACGGCAACAAACCTGGTAAAGAAGATGGTAACAAACCTGGC

>t5269
GAGGAAGACAACAACAAACCTGGTAAAGAAGACAACAACAAGCCTGGCAAAGAAGA
CGGCAACAAGCCTGGTAAAGAAGACAACAACAAACCTGGTAAAGAAGACAACAACA
AACCTGGTAAAGAAGACAACAAAAAACCTGGTAAAGAAGACAACAAAAAACCTGGT
AAAGAAGATGGCAACAAGCCTGGCAAAGAAGACAACAACAAACCTGGT

>t5983

GAGGAAGACAACAACAAACCTGGTAAAGAAGACGGCAACAAACCTGGCAAAGAAGA
CAACAACAAACCTGGTAAAGAAGATGGCAACAAACCTGGCAAAGAAGACAACAAAA
AGCCTGGCAAAGAAGACGGCAACAAGCCTGGTAAAGAAGATGGCAACAAACCTGGT
AAAGAAGACGGCAACAAGCCTGGTAAAGAAGATGGCAACAAACCTGGTAAAGAAGA
CGGCAACAAACCTGGTAAAGAAGATGGTAACAAACCTGGC

>t664
GAGGAAGACAACAACAAACCTGGTAAAGAAGACGGCAACAAACCTGGCAAGAAGAC
AACAACAAGCCTGGTAAAGAAGACAACAACAAGCCTGGTAAAGAAGACGGCAACAA
GCCTGGTAAAGAAGACAACAACAAACCTGGCAAAGAAGACGGCAACAAGCCTGGTA
AAGAAGACAACAACAAGCCTGGTAAAGAAGACGGCAACAAGCCTGGT

>t12147
GAGGAAGACAACAAAAAACCTGGTAAAGAAGACAACAACAAGCCTGGTAAAGAAGA
CAACAACAAGCCTGGCAAAGAAGACGGCAACAAGCCTGGTAAAGAAGACAACAACA
AACCTGGTAAAGAAGACAACAACAAACCTGGTAAAGAAGACAACAAAAAACCTGGT
AAAGAAGACAACAAAAAACCTGGTAAAGAAGACAACAAAAAACCTGGTAAAGAAGA
CAACAAAAAACCTGGTAAAGAAGATGGCAACAAGCCTGGCAAAGAAGACAACAACA
AACCTGGT

>t15796
GAGGAAGACAACAACAAACCTGGTAAAGAAGACAACAACAAGCCTGGTAAAGAAGA
CAACAACAAGCCTGGCAAAGAAGACGGCAACAAGCCTGGTAAAGAAGACAACAACA
AACCTGGTAAAGAAGACAACAACAAACCTGGTAAAGAAGACAACAACAAACCTGGT
AAAGAAGACAACAACAAACCTGGTAAAGAAGACAACAACAAACCTGGTAAAGAAGA
CAACAACAAACCTGGTAAAGAAGATGGCAACAAGCCTGGCAAAGAAGACAACAACA
AACCTGGT

2 Sequencing data of MLST typing in this study

ST59
>arcc19
TTATTAATCCAACAAGCTAAATCGAACAGTGACACAACGCCGGCAATGCCATTGGATAC
TTGTGGTGCAATGTCACAGGGTATGATAGGCTATTGGTTGGAAACTGAAATCAATCGCA
TTTTAACTGAAATGAATAGTGATAGAACTGTAGGCACAATCGTTACACGTGTGGAAGTA
GATAAAGATGATCCACGATTTGATAACCCAACTAAACCAATTGGTCCTTTTTATACGAA
AGAAGAAGTTGAAGAATTACAAAAAGAACAGCCAGACTCAGTATTTAAAGAAGATGC
AGGACGTGGTTATAGAAAAGTAGTTGCGTCACCACTACCTCAATCTATACTAGAACACC
AGTTAATTCGAACTTTAGCAGACGGTAAAAATATTGTCATTGCATGCGGTGGTGGCGGT
ATTCCAGTTATAAAAAAAGAAAATACCTATGAAGGTGTTGAAGCG
>aroe23
AATTTTAATTCTTTAGGATTAGATAATACTTATGAAGCTTTAAATATTCCAATTGAAGATT
TTCATTTAATTAAAGAAATTATTTCAAAAAAAGAATTAGATGGCTTTAATATCACAATTC
CCCATAAAGAACGTATCATACCGTATTTAGATTATGTTGATGAACAAGCGATTAATGCAG

GTGCAGTTAACACTGTTTTGATAAAAGATGGCAAGTGGATAGGGTATAATACAGATGGT
ATCGGTTATGTTAAAGGATTGCACAGCGTTTATCCAGATTTAGAAAATGCATACATTTTA
ATTTTGGGCGCAGGTGGTGCAAGTAAAGGTATTGCTTATGAATTAGCAAAATTTGTAAA
GCCCAAATTAACTGTTGCGAATAGAACGATGGCTCGTTTTGAATCTTGGAATTTAAATAT
AAACCAAATTTCATTGGCAGATGCTGAAAAGTATTTA
>glpf15
GGTGCTGATTGGATTGTCATCACAGCTGGATGGGGATTAGCGGTTACAATGGGTGTGTA
TGCTGTCGGTCAATTCTCAGGTGCACATTTAAACCCAGCGGTGTCTTTAGCTCTTGCAT
TAGACGGAAGTTTTGATTGGTCATTAGTTCCTGGTTATATTGTTGCTCAAATGTTAGGTG
CAATTGTCGGAGCAACGATTGTATGGTTAATGTACTTGCCACATTGGAAAGCGACAGA
AGAAGCTGGCGCGAAATTAGGTGTTTTTTCTACAGCACCGGCTATTAAGAATTACTTTG
CCAACTTTTTAAGTGAGATTATCGGAACAATGGCATTAACTTTAGGTATTTTATTTATCG
GTGTAAACAAAATTGCCGATGGTTTAAATCCTTTAATTGTCGGAGCATTAATTGTTGCA
ATCGGATTAAGTTTAGGCGGTGCTACTGGTTATGCAATCAACCCAGCACGT
>gmk_2
CGAATATTTGAAGATCCAAGTACATCATATAAGTATTCTATTTCAATGACAACACGTCAA
ATGCGTGAAGGTGAAGTTGATGGCGTAGATTACTTTTTTAAAACTAGGGATGCGTTTGA
AGCTTTAATTAAAGATGACCAATTTATAGAATATGCTGAATATGTAGGCAACTATTATGG
TACACCAGTTCAATATGTTAAAGATACAATGGACGAAGGTCATGATGTATTTTTAGAAAT
TGAAGTAGAAGGTGCAAAGCAAGTTAGAAAGAAATTTCCAGATGCGTTATTTATTTTCT
TAGCACCTCCAAGTTTAGATCACTTGAGAGAGCGATTAGTAGGTAGAGGAACAGAATC
TGATGAGAAAATACAAAGTCGTATTAACGAAGCACGTAAAGAAGTCGAAATGATGAAT
TTA
>pta_19
GCAACACAATTACAAGCAACAGATTATGTTACACCAATCGTGTTAGGTGATGAGACTAA
GGTTCAATCTTTAGCGCAAAAACTTAATCTTGATATTTCTAATATTGAATTAATTAATCCT
GCGACAAGTGAATTGAAAGCTGAATTAGTTCAATCATTTGTTGAACGACGTAAAGGGA
AAGCGACTGAAGAACAAGCACAAGAATTATTAAACAATGTGAACTACTTCGGTACAAT
GCTTGTTTATGCTGGTAAAGCAGATGGTCTAGTTAGTGGTGCAGCACATTCAACAGGCG
ACACTGTGCGTCCAGCTTTACAAATCATCAAAACGAAACCAGGTGTATCAAGAACATC
AGGTATCTTCTTTATGATTAAAGGTGATGAACAATACATCTTTGGTGATTGTGCAATCAA
TCCAGAACTTGATTCACAAGGACTTGCAGAAATTGCAGTAGAAAGTGCAAAATCAGCA
TTA
>tpi_20
CACGAAACAGATGAAGAAATTAACAAAAAAGCGCATGCTATTTTCAAACATGGTATGA
CACCAATTATTTGTGTTGGTGAAACAGACGAAGAACGTGAAAGTGGTAAAGCTAACGA
TGTTGTAGGTGAGCAAGTTAAGAAAGCTGTTGCAGGTTTATCTGAAGAGCAACTTAAA
TCAGTTGTAATTGCTTATGAACCAATCTGGGCAATCGGAACTGGTAAATCATCAACATC
TGAAGATGCGAATGAAATGTGTGCATTTGTACGTCAAACTATTTCTGACTTATCAAGCA
AAGAAGTATCAGAAGCAACTCGTATTCAATATGGTGGTAGTGTTAAACCTAACAACATT
AAAGAATACATGGCACAAACTGATATTGATGGGGCATTAGTAGGTGGCGCA
>yqil15
GCGTTTAAAGACGTGCCAGCCTATGATTTAGGTGCGACTTTAATAGAACATATTATTAAA
GAGACGGGTTTGAATCCAAGTGAGATTAATGAAGTCATCATCGGTAACGTACTACAAG

CAGGACAAGGACAAAATCCAGCACGAATTGCTGCTATGAAAGGTGGCTTGCCAGAAA
CAGTACCTGCATTTACAGTGAATAAAGTATGTGGTTCTGGGTTAAAGTCGATTCAATTA
GCATATCAATCTATTGTGACTGGTGAAAATGACATCGTGCTAGCTGGCGGTATGGAGAA
TATGTCTCAATCACCAATGCTTGTCAACAACAGTCGCTTTGGTTTTAAAATGGGACATC
AATCAATGGTTGATAGCATGGTATATGATGGTTTAACAGATGTATTTAATCAATATCATAT
GGGTATTACTGCTGAAAATTTAGTAGAACAATATGGTATTTCAAGAGAAGAACAAGATA
CATTTGCTGTAAACTCACAACAAAAAGCAGTACGTGCACAGCAA

ST338

>arcc19
TTATTAATCCAACAAGCTAAATCGAACAGTGACACAACGCCGGCAATGCCATTGGATAC
TTGTGGTGCAATGTCACAGGGTATGATAGGCTATTGGTTGGAAACTGAAATCAATCGCA
TTTTAACTGAAATGAATAGTGATAGAACTGTAGGCACAATCGTTACACGTGTGGAAGTA
GATAAAGATGATCCACGATTTGATAACCCAACTAAACCAATTGGTCCTTTTTATACGAA
AGAAGAAGTTGAAGAATTACAAAAAGAACAGCCAGACTCAGTATTTAAAGAAGATGC
AGGACGTGGTTATAGAAAAGTAGTTGCGTCACCACTACCTCAATCTATACTAGAACACC
AGTTAATTCGAACTTTAGCAGACGGTAAAAATATTGTCATTGCATGCGGTGGTGGCGGT
ATTCCAGTTATAAAAAAAGAAAATACCTATGAAGGTGTTGAAGCG
>aroe23
AATTTTAATTCTTTAGGATTAGATAATACTTATGAAGCTTTAAATATTCCAATTGAAGATT
TTCATTTAATTAAAGAAATTATTTCAAAAAAAGAATTAGATGGCTTTAATATCACAATTC
CCCATAAAGAACGTATCATACCGTATTTAGATTATGTTGATGAACAAGCGATTAATGCAG
GTGCAGTTAACACTGTTTTGATAAAAGATGGCAAGTGGATAGGGTATAATACAGATGGT
ATCGGTTATGTTAAAGGATTGCACAGCGTTTATCCAGATTTAGAAAATGCATACATTTTA
ATTTTGGGCGCAGGTGGTGCAAGTAAAGGTATTGCTTATGAATTAGCAAAATTTGTAAA
GCCCAAATTAACTGTTGCGAATAGAACGATGGCTCGTTTTGAATCTTGGAATTTAAATAT
AAACCAAATTTCATTGGCAGATGCTGAAAAGTATTTA
>glpf15
GGTGCTGATTGGATTGTCATCACAGCTGGATGGGGATTAGCGGTTACAATGGGTGTGTA
TGCTGTCGGTCAATTCTCAGGTGCACATTTAAACCCAGCGGTGTCTTTAGCTCTTGCAT
TAGACGGAAGTTTTGATTGGTCATTAGTTCCTGGTTATATTGTTGCTCAAATGTTAGGTG
CAATTGTCGGAGCAACGATTGTATGGTTAATGTACTTGCCACATTGGAAAGCGACAGA
AGAAGCTGGCGCGAAATTAGGTGTTTTTTCTACAGCACCGGCTATTAAGAATTACTTTG
CCAACTTTTTAAGTGAGATTATCGGAACAATGGCATTAACTTTAGGTATTTTATTTATCG
GTGTAAACAAAATTGCCGATGGTTTAAATCCTTTAATTGTCGGAGCATTAATTGTTGCA
ATCGGATTAAGTTTAGGCGGTGCTACTGGTTATGCAATCAACCCAGCACGT
>gmk_48
CGAATATTTGAAGATCCAAGTACATCATATAAGTATTCTATTTCAATGACAACACGTCAA
ATGCGTAAAGGTGAAGTTGATGGCGTAGATTACTTTTTTAAAACTAGGGATGCGTTTGA
AGCTTTAATTAAAGATGACCAATTTATAGAATATGCTGAATATGTAGGCAACTATTATGG
TACACCAGTTCAATATGTTAAAGATACAATGGACGAAGGTCATGATGTATTTTTAGAAAT
TGAAGTAGAAGGTGCAAAGCAAGTTAGAAAGAAATTTCCAGATGCGTTATTTATTTTCT
TAGCACCTCCAAGTTTAGATCACTTGAGAGAGCGATTAGTAGGTAGAGGAACAGAATC
TGATGAGAAAATACAAAGTCGTATTAACGAAGCACGTAAAGAAGTCGAAATGATGAAT

TTA
>pta_19
GCAACACAATTACAAGCAACAGATTATGTTACACCAATCGTGTTAGGTGATGAGACTAA
GGTTCAATCTTTAGCGCAAAAACTTAATCTTGATATTTCTAATATTGAATTAATTAATCCT
GCGACAAGTGAATTGAAAGCTGAATTAGTTCAATCATTTGTTGAACGACGTAAAGGGA
AAGCGACTGAAGAACAAGCACAAGAATTATTAAACAATGTGAACTACTTCGGTACAAT
GCTTGTTTATGCTGGTAAAGCAGATGGTCTAGTTAGTGGTGCAGCACATTCAACAGGCG
ACACTGTGCGTCCAGCTTTACAAATCATCAAAACGAAACCAGGTGTATCAAGAACATC
AGGTATCTTCTTTATGATTAAAGGTGATGAACAATACATCTTTGGTGATTGTGCAATCAA
TCCAGAACTTGATTCACAAGGACTTGCAGAAATTGCAGTAGAAAGTGCAAAATCAGCA
TTA
>tpi_20
CACGAAACAGATGAAGAAATTAACAAAAAAGCGCATGCTATTTTCAAACATGGTATGA
CACCAATTATTTGTGTTGGTGAAACAGACGAAGAACGTGAAAGTGGTAAAGCTAACGA
TGTTGTAGGTGAGCAAGTTAAGAAAGCTGTTGCAGGTTTATCTGAAGAGCAACTTAAA
TCAGTTGTAATTGCTTATGAACCAATCTGGGCAATCGGAACTGGTAAATCATCAACATC
TGAAGATGCGAATGAAATGTGTGCATTTGTACGTCAAACTATTTCTGACTTATCAAGCA
AAGAAGTATCAGAAGCAACTCGTATTCAATATGGTGGTAGTGTTAAACCTAACAACATT
AAAGAATACATGGCACAAACTGATATTGATGGGGCATTAGTAGGTGGCGCA
>yqil15
GCGTTTAAAGACGTGCCAGCCTATGATTTAGGTGCGACTTTAATAGAACATATTATTAAA
GAGACGGGTTTGAATCCAAGTGAGATTAATGAAGTCATCATCGGTAACGTACTACAAG
CAGGACAAGGACAAAATCCAGCACGAATTGCTGCTATGAAAGGTGGCTTGCCAGAAA
CAGTACCTGCATTTACAGTGAATAAAGTATGTGGTTCTGGGTTAAAGTCGATTCAATTA
GCATATCAATCTATTGTGACTGGTGAAAATGACATCGTGCTAGCTGGCGGTATGGAGAA
TATGTCTCAATCACCAATGCTTGTCAACAACAGTCGCTTTGGTTTTAAAATGGGACATC
AATCAATGGTTGATAGCATGGTATATGATGGTTTAACAGATGTATTTAATCAATATCATAT
GGGTATTACTGCTGAAAATTTAGTAGAACAATATGGTATTTCAAGAGAAGAACAAGATA
CATTTGCTGTAAACTCACAACAAAAAGCAGTACGTGCACAGCAA

ST1
>arcc1
TTATTAATCCAACAAGCTAAATCGAACAGTGACACAACGCCGGCAATGCCATTGGATAC
TTGTGGTGCAATGTCACAGGGTATGATAGGCTATTGGTTGGAAACTGAAATCAATCGCA
TTTTAACTGAAATGAATAGTGATAGAACTGTAGGCACAATCGTTACACGTGTGGAAGTA
GATAAAGATGATCCACGATTCAATAACCCAACCAAACCAATTGGTCCTTTTTATACGAA
AGAAGAAGTTGAAGAATTACAAAAAGAACAGCCAGACTCAGTCTTTAAAGAAGATGC
AGGACGTGGTTATAGAAAAGTAGTTGCGTCACCACTACCTCAATCTATACTAGAACACC
AGTTAATTCGAACTTTAGCAGACGGTAAAAATATTGTCATTGCATGCGGTGGTGGCGGT
ATTCCAGTTATAAAAAAAGAAAATACCTATGAAGGTGTTGAAGCG
>aroe1
AATTTTAATTCTTTAGGATTAGATGATACTTATGAAGCTTTAAATATTCCAATTGAAGATT
TTCATTTAATTAAAGAAATTATTTCGAAAAAAGAATTAGATGGCTTTAATATCACAATTC
CTCATAAAGAACGTATCATACCGTATTTAGATCATGTTGATGAACAAGCGATTAATGCAG

GTGCAGTTAACACTGTTTTGATAAAAGATGACAAGTGGATAGGGTATAATACAGATGGT
ATTGGTTATGTTAAAGGATTGCACAGCGTTTATCCAGATTTAGAAAATGCATACATTTTA
ATTTTGGGCGCAGGTGGTGCAAGTAAAGGTATTGCTTATGAATTAGCAAAATTTGTAAA
GCCCAAATTAACTGTTGCGAATAGAACGATGGCTCGTTTTGAATCTTGGAATTTAAATAT
AAACCAAATTTCATTAGCAGATGCTGAAAAGTATTTA
>glpf1
GGTGCTGATTGGATTGTCATCACAGCTGGATGGGGATTAGCGGTTACAATGGGTGTGTT
TGCTGTCGGTCAATTCTCAGGTGCACATTTAAACCCAGCGGTGTCTTTAGCTCTTGCAT
TAGACGGAAGTTTTGATTGGTCATTAGTTCCTGGTTATATTGTTGCTCAAATGTTAGGTG
CAATTGTCGGAGCAACAATTGTATGGTTAATGTACTTGCCACATTGGAAAGCGACAGA
AGAAGCTGGCGCGAAATTAGGTGTTTTCTCTACAGCACCGGCTATTAAGAATTACTTTG
CCAACTTTTTAAGTGAGATTATCGGAACAATGGCATTAACTTTAGGTATTTTATTTATCG
GTGTAAACAAAATTGCCGATGGTTTAAATCCTTTAATTGTCGGAGCATTAATTGTTGCA
ATCGGATTAAGTTTAGGCGGTGCTACTGGTTATGCAATCAACCCAGCACGT
>gmk_1
CGAATATTTGAAGATCCAAGTACATCATATAAGTATTCTATTTCAATGACAACACGTCAA
ATGCGTGAAGGTGAAGTTGATGGCGTAGATTACTTTTTTAAAACTAGGGATGCGTTTGA
AGCTTTAATCAAAGATGACCAATTTATAGAATATGCTGAATATGTAGGCAACTATTATGG
TACACCAGTTCAATATGTTAAAGATACAATGGACGAAGGTCATGATGTATTTTTAGAAAT
TGAAGTAGAAGGTGCAAAGCAAGTTAGAAAGAAATTTCCAGATGCGCTATTTATTTTCT
TAGCACCTCCAAGTTTAGAACACTTGAGAGAGCGATTAGTAGGTAGAGGAACAGAATC
TGATGAGAAAATACAAAGTCGTATTAACGAAGCGCGTAAAGAAGTTGAAATGATGAAT
TTA
>pta_1
GCAACACAATTACAAGCAACAGATTATGTTACACCAATCGTGTTAGGTGATGAGACTAA
GGTTCAATCTTTAGCGCAAAAACTTGATCTTGATATTTCTAATATTGAATTAATTAATCCT
GCGACAAGTGAATTGAAAGCTGAATTAGTTCAATCATTTGTTGAACGACGTAAAGGTA
AAGCGACTGAAGAACAAGCACAAGAATTATTAAACAATGTGAACTACTTCGGTACAAT
GCTTGTTTATGCTGGTAAAGCAGATGGTTTAGTTAGTGGTGCAGCACATTCAACAGGAG
ACACTGTGCGTCCAGCTTTACAAATCATCAAAACGAAACCAGGTGTATCAAGAACATC
AGGTATCTTCTTTATGATTAAAGGTGATGTACAATACATCTTTGGTGATTGTGCAATCAA
TCCAGAACTTGATTCACAAGGACTTGCAGAAATTGCAGTAGAAAGTGCAAAATCAGCA
TTA
>tpi_1
CACGAAACAGATGAAGAAATTAACAAAAAAGCGCACGCTATTTTCAAACATGGAATGA
CTCCAATTATTTGTGTTGGTGAAACAGACGAAGAGCGTGAAAGTGGTAAAGCTAACGA
TGTTGTAGGTGAGCAAGTTAAGAAAGCTGTTGCAGGTTTATCTGAAGATCAACTTAAAT
CAGTTGTAATTGCTTATGAGCCAATCTGGGCAATCGGAACTGGTAAATCATCAACATCT
GAAGATGCAAATGAAATGTGTGCATTTGTACGTCAAACTATTGCTGACTTATCAAGCAA
AGAAGTATCAGAAGCAACTCGTATTCAATATGGTGGTAGTGTTAAACCTAACAACATTA
AAGAATACATGGCACAAACTGATATTGATGGGGCATTAGTAGGTGGCGCA
>yqil1
GCGTTTAAAGACGTGCCAGCCTATGATTTAGGTGCGACTTTAATAGAACATATTATTAAA
GAGACGGGTTTGAATCCAAGTGAGATTGATGAAGTTATCATCGGTAACGTACTACAAG

CAGGACAAGGACAAAATCCAGCACGAATTGCTGCTATGAAAGGTGGCTTGCCAGAAA
CAGTACCTGCATTTACAGTGAATAAAGTATGTGGTTCTGGGTTAAAGTCGATTCAATTA
GCATATCAATCTATTGTGACTGGTGAAAATGACATCGTGCTAGCTGGCGGTATGGAGAA
TATGTCTCAGTCACCAATGCTTGTCAACAACAGTCGCTTCGGTTTTAAAATGGGACATC
AATCAATGGTTGATAGCATGGTATATGATGGTTTAACAGATGTATTTAATCAATATCATAT
GGGTATTACTGCTGAAAATTTAGTGGAGCAATATGGTATTTCAAGAGAAGAACAAGATA
CATTTGCTGTAAACTCACAACAAAAAGCAGTACGTGCACAGCAA

ST188

>arcc3
TTATTAATCCAACAAGCTAAATCGAACAGTGACACAACGCCGGCAATGCCATTGGATAC
TTGTGGTGCAATGTCACAGGGTATGATAGGCTATTGGTTGGAAACTGAAATCAATCGCA
TTTTAACTGAAATGAATAGTGATAGAACTGTAGGCACAATCGTTACACGTGTGGAAGTA
GATAAAGATGATCCACGATTTGATAACCCAACTAAACCAATTGGTCCTTTTTATACGAA
AGAAGAAGTTGAAGAATTACAAAAAGAACAGCCAGACTCAGTCTTTAAAGAAGATGC
AGGACGTGGTTATAGAAAAGTAGTTGCGTCACCACTACCTCAATCTATACTAGAACACC
AGTTAATTCGAACTTTAGCAGACGGTAAAAATATTGTCATTGCATGCGGTGGTGGCGGT
ATTCCAGTTATAAAAAAAGAAAATACCTATGAAGGTGTTGAAGCG

>aroe1
AATTTAATTCTTTAGGATTAGATGATACTTATGAAGCTTTAAATATTCCAATTGAAGATT
TTCATTTAATTAAAGAAATTATTTCGAAAAAAGAATTAGATGGCTTTAATATCACAATTC
CTCATAAAGAACGTATCATACCGTATTTAGATCATGTTGATGAACAAGCGATTAATGCAG
GTGCAGTTAACACTGTTTTGATAAAAGATGACAAGTGGATAGGGTATAATACAGATGGT
ATTGGTTATGTTAAAGGATTGCACAGCGTTTATCCAGATTTAGAAAATGCATACATTTTA
ATTTTGGGCGCAGGTGGTGCAAGTAAAGGTATTGCTTATGAATTAGCAAAATTTGTAAA
GCCCAAATTAACTGTTGCGAATAGAACGATGGCTCGTTTTGAATCTTGGAATTTAAATAT
AAACCAAATTTCATTAGCAGATGCTGAAAAGTATTTA

>glpf1
GGTGCTGATTGGATTGTCATCACAGCTGGATGGGGATTAGCGGTTACAATGGGTGTGTT
TGCTGTCGGTCAATTCTCAGGTGCACATTTAAACCCAGCGGTGTCTTTAGCTCTTGCAT
TAGACGGAAGTTTTGATTGGTCATTAGTTCCTGGTTATATTGTTGCTCAAATGTTAGGTG
CAATTGTCGGAGCAACAATTGTATGGTTAATGTACTTGCCACATTGGAAAGCGACAGA
AGAAGCTGGCGCGAAATTAGGTGTTTTCTCTACAGCACCGGCTATTAAGAATTACTTTG
CCAACTTTTTAAGTGAGATTATCGGAACAATGGCATTAACTTTAGGTATTTTATTTATCG
GTGTAAACAAAATTGCCGATGGTTTAAATCCTTTAATTGTCGGAGCATTAATTGTTGCA
ATCGGATTAAGTTTAGGCGGTGCTACTGGTTATGCAATCAACCCAGCACGT

>gmk_8
CGAATATTTGAAGATCCAAGTACATCGTATAAGTATTCTATTTCAATGACAACACGTCAA
ATGCGTGAAGGTGAAGTTGATGGCGTAGATTACTTTTTTAAAACTAGGGATGCGTTTGA
AGCTTTAATTAAAGATGACCAATTTATAGAATATGCTGAATATGTAGGCAACTATTATGG
TACACCAGTTCAATATGTTAAAGATACAATGGACGAAGGTCATGATGTATTTTTAGAAAT
TGAAGTAGAAGGTGCAAAGCAAGTTAGAAAGAAATTTCCAGATGCGTTATTTATTTTCT
TAGCACCTCCAAGTTTAGATCACTTGAGAGAGCGATTAGTAGGTAGAGGAACAGAATC
CAATGAGAAAATACAAAGTCGTATTAACGAAGCGCGTAAAGAAGTTGAAATGATGAAT

TTA
>pta_1
GCAACACAATTACAAGCAACAGATTATGTTACACCAATCGTGTTAGGTGATGAGACTAA
GGTTCAATCTTTAGCGCAAAAACTTGATCTTGATATTTCTAATATTGAATTAATTAATCCT
GCGACAAGTGAATTGAAAGCTGAATTAGTTCAATCATTTGTTGAACGACGTAAAGGTA
AAGCGACTGAAGAACAAGCACAAGAATTATTAAACAATGTGAACTACTTCGGTACAAT
GCTTGTTTATGCTGGTAAAGCAGATGGTTTAGTTAGTGGTGCAGCACATTCAACAGGAG
ACACTGTGCGTCCAGCTTTACAAATCATCAAAACGAAACCAGGTGTATCAAGAACATC
AGGTATCTTCTTTATGATTAAAGGTGATGTACAATACATCTTTGGTGATTGTGCAATCAA
TCCAGAACTTGATTCACAAGGACTTGCAGAAATTGCAGTAGAAAGTGCAAAATCAGCA
TTA
>tpi_1
CACGAAACAGATGAAGAAATTAACAAAAAAGCGCACGCTATTTTCAAACATGGAATGA
CTCCAATTATTTGTGTTGGTGAAACAGACGAAGAGCGTGAAAGTGGTAAAGCTAACGA
TGTTGTAGGTGAGCAAGTTAAGAAAGCTGTTGCAGGTTTATCTGAAGATCAACTTAAAT
CAGTTGTAATTGCTTATGAGCCAATCTGGGCAATCGGAACTGGTAAATCATCAACATCT
GAAGATGCAAATGAAATGTGTGCATTTGTACGTCAAACTATTGCTGACTTATCAAGCAA
AGAAGTATCAGAAGCAACTCGTATTCAATATGGTGGTAGTGTTAAACCTAACAACATTA
AAGAATACATGGCACAAACTGATATTGATGGGGCATTAGTAGGTGGCGCA
>yqil1
GCGTTTAAAGACGTGCCAGCCTATGATTTAGGTGCGACTTTAATAGAACATATTATTAAA
GAGACGGGTTTGAATCCAAGTGAGATTGATGAAGTTATCATCGGTAACGTACTACAAG
CAGGACAAGGACAAAATCCAGCACGAATTGCTGCTATGAAAGGTGGCTTGCCAGAAA
CAGTACCTGCATTTACAGTGAATAAAGTATGTGGTTCTGGGTTAAAGTCGATTCAATTA
GCATATCAATCTATTGTGACTGGTGAAAATGACATCGTGCTAGCTGGCGGTATGGAGAA
TATGTCTCAGTCACCAATGCTTGTCAACAACAGTCGCTTCGGTTTTAAAATGGGACATC
AATCAATGGTTGATAGCATGGTATATGATGGTTTAACAGATGTATTTAATCAATATCATAT
GGGTATTACTGCTGAAAATTTAGTGGAGCAATATGGTATTTCAAGAGAAGAACAAGATA
CATTTGCTGTAAACTCACAACAAAAAGCAGTACGTGCACAGCAA

ST5
>arcc1
TTATTAATCCAACAAGCTAAATCGAACAGTGACACAACGCCGGCAATGCCATTGGATAC
TTGTGGTGCAATGTCACAGGGTATGATAGGCTATTGGTTGGAAACTGAAATCAATCGCA
TTTTAACTGAAATGAATAGTGATAGAACTGTAGGCACAATCGTTACACGTGTGGAAGTA
GATAAAGATGATCCACGATTCAATAACCCAACCAAACCAATTGGTCCTTTTTATACGAA
AGAAGAAGTTGAAGAATTACAAAAAGAACAGCCAGACTCAGTCTTTAAAGAAGATGC
AGGACGTGGTTATAGAAAAGTAGTTGCGTCACCACTACCTCAATCTATACTAGAACACC
AGTTAATTCGAACTTTAGCAGACGGTAAAAATATTGTCATTGCATGCGGTGGTGGCGGT
ATTCCAGTTATAAAAAAAGAAAATACCTATGAAGGTGTTGAAGCG
>aroe4
AATTTTAATTCTTTAGGATTAGATGATACTTATGAAGCTTTAAATATTCCAATTGAAGATT
TTCATTTAATTAAAGAAATTATTTCGAAAAAAGAATTAGATGGCTTTAATATCACAATTC
CTCATAAAGAACGTATCATACCGTATTTAGATTATGTTGATGAACAAGCGATTAATGCAG

GTGCAGTTAACACTGTTTTGATAAAAGATGGCAAGTGGATAGGGTATAATACAGATGGT
ATTGGTTATGTTAAAGGATTGCACAGCGTTTATCCAGATTTAGAAAATGCATACATTTTA
ATTTTGGGCGCAGGTGGTGCAAGTAAAGGTATTGCTTATGAATTAGCAAAATTTGTAAA
GCCCAAATTAACTGTTGCGAATAGAACGATGGCTCGTTTTGAATCTTGGAATTTAAATAT
AAACCAAATTTCATTGGCAGATGCTGAAAAGTATTTA
>glpf1
GGTGCTGATTGGATTGTCATCACAGCTGGATGGGGATTAGCGGTTACAATGGGTGTGTT
TGCTGTCGGTCAATTCTCAGGTGCACATTTAAACCCAGCGGTGTCTTTAGCTCTTGCAT
TAGACGGAAGTTTTGATTGGTCATTAGTTCCTGGTTATATTGTTGCTCAAATGTTAGGTG
CAATTGTCGGAGCAACAATTGTATGGTTAATGTACTTGCCACATTGGAAAGCGACAGA
AGAAGCTGGCGCGAAATTAGGTGTTTTCTCTACAGCACCGGCTATTAAGAATTACTTTG
CCAACTTTTTAAGTGAGATTATCGGAACAATGGCATTAACTTTAGGTATTTTATTTATCG
GTGTAAACAAAATTGCCGATGGTTTAAATCCTTTAATTGTCGGAGCATTAATTGTTGCA
ATCGGATTAAGTTTAGGCGGTGCTACTGGTTATGCAATCAACCCAGCACGT
>gmk_4
CGAATATTTGAAGATCCAAGTACATCATATAAGTATTCTATTTCAATGACAACACGTCAA
ATGCGTGAAGGTGAAGTTGATGGCGTAGATTACTTTTTTAAAACTAGGGATGCGTTTGA
AGCTTTAATTAAAGATGACCAATTTATAGAATATGCTGAATATGTAGGCAACTATTATGG
TACACCAGTTCAATATGTTAAAGATACAATGGACGAAGGTCATGATGTATTTTTAGAAAT
TGAAGTAGAAGGTGCAAAGCAAGTTAGAAAGAAATTTCCAGATGCGTTATTTATTTTCT
TAGCACCTCCAAGTTTAGATCACTTGAGAGAGCGATTAGTAGGTAGAGGAACAGAATC
CAATGAGAAAATACAAAGTCGTATTAACGAAGCGCGTAAAGAAGTTGAAATGATGAAT
TTA
>pta_12
GCAACACAATTACAAGCAACAGATTATGTTACACCAATCGTGTTAGGTGATGAGACTAA
GGTTCAATCTTTAGCGCAAAAACTTGATCTTGATATTTCTAATATTGAATTAATTAATCCT
GCGACAAGTGAATTGAAAGCTGAATTAGTTCAATCATTTGTTGAACGACGTAAAGGTA
AAGCGACTGAAGAACAAGCACAAGAATTATTAAACAATGTGAACTACTTCGGTACAAT
GCTTGTTTATGCTGGTAAAGCAGATGGTTTAGTTAGTGGTGCAGCACATTCAACAGGAG
ACACTGTGCGTCCAGCTTTACAAATCATCAAAACGAAACCAGGTGTATCAAGAACATC
AGGTATCTTCTTTATGATTAAAGGTGATGAACAATACATCTTTGGTGATTGTGCAATCAA
TCCAGAACTTGATTCACAAGGACTTGCAGAAATTGCAGTAGAAAGTGCAAAATCAGCA
TTA
>tpi_1
CACGAAACAGATGAAGAAATTAACAAAAAAGCGCACGCTATTTTCAAACATGGAATGA
CTCCAATTATTTGTGTTGGTGAAACAGACGAAGAGCGTGAAAGTGGTAAAGCTAACGA
TGTTGTAGGTGAGCAAGTTAAGAAAGCTGTTGCAGGTTTATCTGAAGATCAACTTAAAT
CAGTTGTAATTGCTTATGAGCCAATCTGGGCAATCGGAACTGGTAAATCATCAACATCT
GAAGATGCAAATGAAATGTGTGCATTTGTACGTCAAACTATTGCTGACTTATCAAGCAA
AGAAGTATCAGAAGCAACTCGTATTCAATATGGTGGTAGTGTTAAACCTAACAACATTA
AAGAATACATGGCACAAACTGATATTGATGGGGCATTAGTAGGTGGCGCA
>yqil10
GCGTTTAAAGACGTGCCAGCCTATGATTTAGGTGCGACTTTAATAGAACATATTATTAAA
GAGACGGGTTTGAATCCAAGTGAGATTGATGAAGTTATCATCGGTAACGTACTACAAG

CAGGACAAGGACAAAATCCAGCACGAATTGCTGCTATGAAAGGTGGCTTGCCAGAAA
CAGTACCTGCATTTACAGTGAATAAAGTATGTGGTTCTGGGTTAAAGTCGATTCAATTA
GCATATCAATCTATTGTGACTGGTGAAAATGACATCGTGCTAGCTGGCGGTATGGAGAA
TATGTCTCAGTCACCAATGCTTGTCAACAACAGTCGCTTCGGTTTTAAAATGGGACATC
AATCAATGGTTGATAGCATGGTATATGATGGTTTAACAGATGTATTTAATCAATATCATAT
GGGTATTACTGCTGAAAATTTAGTAGAGCAATATGGTATTTCAAGAGAAGAACAAGATA
CATTTGCTGTAAACTCACAACATAAAGCAGTACGTGCACAGCAA

ST1507

>arcc1
TTATTAATCCAACAAGCTAAATCGAACAGTGACACAACGCCGGCAATGCCATTGGATAC
TTGTGGTGCAATGTCACAGGGTATGATAGGCTATTGGTTGGAAACTGAAATCAATCGCA
TTTTAACTGAAATGAATAGTGATAGAACTGTAGGCACAATCGTTACACGTGTGGAAGTA
GATAAAGATGATCCACGATTCAATAACCCAACCAAACCAATTGGTCCTTTTTATACGAA
AGAAGAAGTTGAAGAATTACAAAAAGAACAGCCAGACTCAGTCTTTAAAGAAGATGC
AGGACGTGGTTATAGAAAAGTAGTTGCGTCACCACTACCTCAATCTATACTAGAACACC
AGTTAATTCGAACTTTAGCAGACGGTAAAAATATTGTCATTGCATGCGGTGGTGGCGGT
ATTCCAGTTATAAAAAAAGAAAATACCTATGAAGGTGTTGAAGCG
>aroe4
AATTTTAATTCTTTAGGATTAGATGATACTTATGAAGCTTTAAATATTCCAATTGAAGATT
TTCATTTAATTAAAGAAATTATTTCGAAAAAAGAATTAGATGGCTTTAATATCACAATTC
CTCATAAAGAACGTATCATACCGTATTTAGATTATGTTGATGAACAAGCGATTAATGCAG
GTGCAGTTAACACTGTTTTGATAAAAGATGGCAAGTGGATAGGGTATAATACAGATGGT
ATTGGTTATGTTAAAGGATTGCACAGCGTTTATCCAGATTTAGAAAATGCATACATTTTA
ATTTTGGGCGCAGGTGGTGCAAGTAAAGGTATTGCTTATGAATTAGCAAAATTTGTAAA
GCCCAAATTAACTGTTGCGAATAGAACGATGGCTCGTTTTGAATCTTGGAATTTAAATAT
AAACCAAATTTCATTGGCAGATGCTGAAAAGTATTTA
>glpf1
GGTGCTGATTGGATTGTCATCACAGCTGGATGGGGATTAGCGGTTACAATGGGTGTGTT
TGCTGTCGGTCAATTCTCAGGTGCACATTTAAACCCAGCGGTGTCTTTAGCTCTTGCAT
TAGACGGAAGTTTTGATTGGTCATTAGTTCCTGGTTATATTGTTGCTCAAATGTTAGGTG
CAATTGTCGGAGCAACAATTGTATGGTTAATGTACTTGCCACATTGGAAAGCGACAGA
AGAAGCTGGCGCGAAATTAGGTGTTTTCTCTACAGCACCGGCTATTAAGAATTACTTTG
CCAACTTTTTAAGTGAGATTATCGGAACAATGGCATTAACTTTAGGTATTTTATTTATCG
GTGTAAACAAAATTGCCGATGGTTTAAATCCTTTAATTGTCGGAGCATTAATTGTTGCA
ATCGGATTAAGTTTAGGCGGTGCTACTGGTTATGCAATCAACCCAGCACGT
>gmk_4
CGAATATTTGAAGATCCAAGTACATCATATAAGTATTCTATTTCAATGACAACACGTCAA
ATGCGTGAAGGTGAAGTTGATGGCGTAGATTACTTTTTTAAAACTAGGGATGCGTTTGA
AGCTTTAATTAAAGATGACCAATTTATAGAATATGCTGAATATGTAGGCAACTATTATGG
TACACCAGTTCAATATGTTAAAGATACAATGGACGAAGGTCATGATGTATTTTTAGAAAT
TGAAGTAGAAGGTGCAAAGCAAGTTAGAAAGAAATTTCCAGATGCGTTATTTATTTTCT
TAGCACCTCCAAGTTTAGATCACTTGAGAGAGCGATTAGTAGGTAGAGGAACAGAATC
CAATGAGAAAATACAAAGTCGTATTAACGAAGCGCGTAAAGAAGTTGAAATGATGAAT

TTA
>pta_4
GCAACACAATTACAAGCAACAGATTATGTTACACCAATCGTGTTAGGTGATGAGACTAA
GGTTCAATCTTTAGCGCAAAAACTTGATCTTGATATTTCTAATATTGAATTAATTAATCCT
GCGACAAGTGAATTGAAAGCTGAATTAGTTCAATCATTTGTTGAACGACGTAAAGGTA
AAGCGACTGAAGAACAAGCACAAGAATTATTAAACAATGTGAACTACTTCGGTACAAT
GCTTGTTTATGCTGGTAAAGCAGATGGTTTAGTTAGTGGTGCAGCACATTCAACAGGCG
ACACTGTGCGTCCAGCTTTACAAATCATCAAAACGAAACCAGGTGTATCAAGAACATC
AGGTATCTTCTTTATGATTAAAGGTGATGAACAATACATCTTTGGTGATTGTGCAATCAA
TCCAGAACTTGATTCACAAGGACTTGCAGAAATTGCAGTAGAAAGTGCAAAATCAGCA
TTA
>tpi_4
CACGAAACAGATGAAGAAATTAACAAAAAAGCGCACGCTATTTTCAAACATGGAATGA
CTCCAATTATATGTGTTGGTGAAACAGACGAAGAGCGTGAAAGTGGTAAAGCTAACGA
TGTTGTAGGTGAGCAAGTTAAGAAAGCTGTTGCAGGTTTATCTGAAGATCAACTTAAAT
CAGTTGTAATTGCTTATGAACCAATCTGGGCAATCGGAACTGGTAAATCATCAACATCT
GAAGATGCAAATGAAATGTGTGCATTTGTACGTCAAACTATTGCTGACTTATCAAGCAA
AGAAGTATCAGAAGCAACTCGTATTCAATATGGTGGTAGTGTTAAACCTAACAACATTA
AAGAATACATGGCACAAACTGATATTGATGGGGCATTAGTAGGTGGCGCA
>yqil3
GCGTTTAAAGACGTGCCAGCCTATGATTTAGGTGCGACTTTAATAGAACATATTATTAAA
GAGACGGGTTTGAATCCAAGTGAGATTGATGAAGTTATCATCGGTAACGTACTACAAG
CAGGACAAGGACAAAATCCAGCACGAATTGCTGCTATGAAAGGTGGCTTGCCAGAAA
CAGTACCTGCATTTACGGTGAATAAAGTATGTGGTTCTGGGTTAAAGTCGATTCAATTA
GCATATCAATCTATTGTGACTGGTGAAAATGACATCGTGCTAGCTGGCGGTATGGAGAA
TATGTCTCAATCACCAATGCTTGTCAACAACAGTCGCTTTGGTTTTAAAATGGGACATC
AATCAATGGTTGATAGCATGGTATATGATGGTTTAACAGATGTATTTAATCAATATCATAT
GGGTATTACTGCTGAAAATTTAGTAGAGCAATATGGTATTTCAAGAGAAGAACAAGATA
CATTTGCTGTAAACTCACAACAAAAAGCAGTACGTGCACAGCAA

ST149

>arcc1
TTATTAATCCAACAAGCTAAATCGAACAGTGACACAACGCCGGCAATGCCATTGGATAC
TTGTGGTGCAATGTCACAGGGTATGATAGGCTATTGGTTGGAAACTGAAATCAATCGCA
TTTTAACTGAAATGAATAGTGATAGAACTGTAGGCACAATCGTTACACGTGTGGAAGTA
GATAAAGATGATCCACGATTCAATAACCCAACCAAACCAATTGGTCCTTTTTATACGAA
AGAAGAAGTTGAAGAATTACAAAAAGAACAGCCAGACTCAGTCTTTAAAGAAGATGC
AGGACGTGGTTATAGAAAAGTAGTTGCGTCACCACTACCTCAATCTATACTAGAACACC
AGTTAATTCGAACTTTAGCAGACGGTAAAAATATTGTCATTGCATGCGGTGGTGGCGGT
ATTCCAGTTATAAAAAAAGAAAATACCTATGAAGGTGTTGAAGCG
>aroe4
AATTTTAATTCTTTAGGATTAGATGATACTTATGAAGCTTTAAATATTCCAATTGAAGATT
TTCATTTAATTAAAGAAATTATTTCGAAAAAAGAATTAGATGGCTTTAATATCACAATTC
CTCATAAAGAACGTATCATACCGTATTTAGATTATGTTGATGAACAAGCGATTAATGCAG

GTGCAGTTAACACTGTTTTGATAAAAGATGGCAAGTGGATAGGGTATAATACAGATGGT
ATTGGTTATGTTAAAGGATTGCACAGCGTTTATCCAGATTTAGAAAATGCATACATTTTA
ATTTTGGGCGCAGGTGGTGCAAGTAAAGGTATTGCTTATGAATTAGCAAAATTTGTAAA
GCCCAAATTAACTGTTGCGAATAGAACGATGGCTCGTTTTGAATCTTGGAATTTAAATAT
AAACCAAATTTCATTGGCAGATGCTGAAAAGTATTTA
>glpf1
GGTGCTGATTGGATTGTCATCACAGCTGGATGGGGATTAGCGGTTACAATGGGTGTGTT
TGCTGTCGGTCAATTCTCAGGTGCACATTTAAACCCAGCGGTGTCTTTAGCTCTTGCAT
TAGACGGAAGTTTTGATTGGTCATTAGTTCCTGGTTATATTGTTGCTCAAATGTTAGGTG
CAATTGTCGGAGCAACAATTGTATGGTTAATGTACTTGCCACATTGGAAAGCGACAGA
AGAAGCTGGCGCGAAATTAGGTGTTTTCTCTACAGCACCGGCTATTAAGAATTACTTTG
CCAACTTTTTAAGTGAGATTATCGGAACAATGGCATTAACTTTAGGTATTTTATTTATCG
GTGTAAACAAAATTGCCGATGGTTTAAATCCTTTAATTGTCGGAGCATTAATTGTTGCA
ATCGGATTAAGTTTAGGCGGTGCTACTGGTTATGCAATCAACCCAGCACGT
>gmk_4
CGAATATTTGAAGATCCAAGTACATCATATAAGTATTCTATTTCAATGACAACACGTCAA
ATGCGTGAAGGTGAAGTTGATGGCGTAGATTACTTTTTTAAAACTAGGGATGCGTTTGA
AGCTTTAATTAAAGATGACCAATTTATAGAATATGCTGAATATGTAGGCAACTATTATGG
TACACCAGTTCAATATGTTAAAGATACAATGGACGAAGGTCATGATGTATTTTTAGAAAT
TGAAGTAGAAGGTGCAAAGCAAGTTAGAAAGAAATTTCCAGATGCGTTATTTATTTTCT
TAGCACCTCCAAGTTTAGATCACTTGAGAGAGCGATTAGTAGGTAGAGGAACAGAATC
CAATGAGAAAATACAAAGTCGTATTAACGAAGCGCGTAAAGAAGTTGAAATGATGAAT
TTA
>pta_12
GCAACACAATTACAAGCAACAGATTATGTTACACCAATCGTGTTAGGTGATGAGACTAA
GGTTCAATCTTTAGCGCAAAAACTTGATCTTGATATTTCTAATATTGAATTAATTAATCCT
GCGACAAGTGAATTGAAAGCTGAATTAGTTCAATCATTTGTTGAACGACGTAAAGGTA
AAGCGACTGAAGAACAAGCACAAGAATTATTAAACAATGTGAACTACTTCGGTACAAT
GCTTGTTTATGCTGGTAAAGCAGATGGTTTAGTTAGTGGTGCAGCACATTCAACAGGAG
ACACTGTGCGTCCAGCTTTACAAATCATCAAAACGAAACCAGGTGTATCAAGAACATC
AGGTATCTTCTTTATGATTAAAGGTGATGAACAATACATCTTTGGTGATTGTGCAATCAA
TCCAGAACTTGATTCACAAGGACTTGCAGAAATTGCAGTAGAAAGTGCAAAATCAGCA
TTA
>tpi_41
CACGAAACAGATGAAAAAATTAACAAAAAAGCGCACGCTATTTTCAAACATGGAATGA
CTCCAATTATTTGTGTTGGTGAAACAGACGAAGAGCGTGAAAGTGGTAAAGCTAACGA
TGTTGTAGGTGAGCAAGTTAAGAAAGCTGTTGCAGGTTTATCTGAAGATCAACTTAAAT
CAGTTGTAATTGCTTATGAGCCAATCTGGGCAATCGGAACTGGTAAATCATCAACATCT
GAAGATGCAAATGAAATGTGTGCATTTGTACGTCAAACTATTGCTGACTTATCAAGCAA
AGAAGTATCAGAAGCAACTCGTATTCAATATGGTGGTAGTGTTAAACCTAACAACATTA
AAGAATACATGGCACAAACTGATATTGATGGGGCATTAGTAGGTGGCGCA
>yqil10
GCGTTTAAAGACGTGCCAGCCTATGATTTAGGTGCGACTTTAATAGAACATATTATTAAA
GAGACGGGTTTGAATCCAAGTGAGATTGATGAAGTTATCATCGGTAACGTACTACAAG

CAGGACAAGGACAAAATCCAGCACGAATTGCTGCTATGAAAGGTGGCTTGCCAGAAA
CAGTACCTGCATTTACAGTGAATAAAGTATGTGGTTCTGGGTTAAAGTCGATTCAATTA
GCATATCAATCTATTGTGACTGGTGAAAATGACATCGTGCTAGCTGGCGGTATGGAGAA
TATGTCTCAGTCACCAATGCTTGTCAACAACAGTCGCTTCGGTTTTAAAATGGGACATC
AATCAATGGTTGATAGCATGGTATATGATGGTTTAACAGATGTATTTAATCAATATCATAT
GGGTATTACTGCTGAAAATTTAGTAGAGCAATATGGTATTTCAAGAGAAGAACAAGATA
CATTTGCTGTAAACTCACAACATAAAGCAGTACGTGCACAGCAA

ST398

>arcc3
TTATTAATCCAACAAGCTAAATCGAACAGTGACACAACGCCGGCAATGCCATTGGATAC
TTGTGGTGCAATGTCACAGGGTATGATAGGCTATTGGTTGGAAACTGAAATCAATCGCA
TTTTAACTGAAATGAATAGTGATAGAACTGTAGGCACAATCGTTACACGTGTGGAAGTA
GATAAAGATGATCCACGATTTGATAACCCAACTAAACCAATTGGTCCTTTTTATACGAA
AGAAGAAGTTGAAGAATTACAAAAAGAACAGCCAGACTCAGTCTTTAAAGAAGATGC
AGGACGTGGTTATAGAAAAGTAGTTGCGTCACCACTACCTCAATCTATACTAGAACACC
AGTTAATTCGAACTTTAGCAGACGGTAAAAATATTGTCATTGCATGCGGTGGTGGCGGT
ATTCCAGTTATAAAAAAAGAAAATACCTATGAAGGTGTTGAAGCG
>aroe35
AATTTAATTCTTTGGGATTAGATGATAGTTATGAAGCTTTAAATATTCCAATTGAAGATT
TTCATTTAATTAAAGAAATTATTTCAAAAAAAGAATTAGATGGCTTTAATATCACAATTC
CTCATAAAGAGCGTATCATACCGTATTTAGATCATGTTGATGAACAAGCGATTAATGCAG
GTGCAGTTAATACTGTTTTGATAAAAGATGGCAAGTGGATAGGGTATAATACAGATGGT
ATTGGTTATGTAAAAGGATTGCACAGCGTTTATCCAGATTTAGAAAATGCATACATTTTA
ATTTTGGGAGCAGGTGGTGCAAGTAAAGGTATTGCTTATGAATTAGCAAAATTTGTAAA
GCCCAAATTAACTGTTGCGAATAGAACGTTGGCTCGTTTTGAATCTTGGAATTTAAATAT
AAATCAAATTTCATTGGCAGATGCTGAAAAGTATTTA
>glpf19
GGTGCTGATTGGATTGTCATCACAGCTGGATGGGGATTAGCGGTTACAATGGGTGTGTA
TGCTGTCGGTCAATTCTCAGGTGCACATTTAAACCCAGCGGTGTCTTTAGCTCTTGCAT
TAGACGGAAGTTTTGATTGGTCATTAGTTCCTGGTTATATTGTTGCTCAAATGTTAGGTG
CAATTGTCGGAGCAACAATTGTATGGTTAATGTACTTGCCACATTGGAAAGCGACAGA
AGAAGCTGGCGCGAAATTAGGTGTTTTCTCTACAGCACCAGCTATTAAGAATTACTTTG
CCAACTTTTTAAGTGAGATTATCGGAACAATGGCATTAACTTTAGGTATTTTATTTATCG
GTGTAAACAAAATTGCCGATGGTTTAAATCCTTTAATTGTCGGAGCATTAATTGTTGCA
ATCGGATTAAGTTTAGGCGGTGCTACTGGTTATGCAATCAACCCAGCACGT
>gmk_2
CGAATATTTGAAGATCCAAGTACATCATATAAGTATTCTATTTCAATGACAACACGTCAA
ATGCGTGAAGGTGAAGTTGATGGCGTAGATTACTTTTTTAAAACTAGGGATGCGTTTGA
AGCTTTAATTAAAGATGACCAATTTATAGAATATGCTGAATATGTAGGCAACTATTATGG
TACACCAGTTCAATATGTTAAAGATACAATGGACGAAGGTCATGATGTATTTTTAGAAAT
TGAAGTAGAAGGTGCAAAGCAAGTTAGAAAGAAATTTCCAGATGCGTTATTTATTTTCT
TAGCACCTCCAAGTTTAGATCACTTGAGAGAGCGATTAGTAGGTAGAGGAACAGAATC
TGATGAGAAAATACAAAGTCGTATTAACGAAGCACGTAAAGAAGTCGAAATGATGAAT

TTA
>pta_20
GCAACACAATTACAAGCAACAGATTATGTTACACCAATCGTGTTAGGTGATGAGACTAA
GGTTCAATCTTTAGCGCAAAAACTTAATCTTGATATTTCTAATATTGAATTAATTAATCCT
GCGACAAGTGAATTGAAAGCTGAATTAGTTCAATCATTTGTTGAACGACGTAAAGGTA
AAGCGACTGAAGAACAAGCACAAGAATTATTAAACAATGTGAACTACTTCGGTACAAT
GCTTGTTTATGCTGGTAAAGCAGATGGCTTAGTTAGTGGTGCAGCACATTCAACAGGCG
ACACTGTGCGTCCAGCATTACAAATCATCAAAACGAAACCAGGTGTATCAAGAACATC
AGGTATCTTCTTTATGATTAAAGGTGATGAACAATACATCTTTGGTGATTGTGCAATCAA
TCCAGAACTTGATTCACAAGGACTTGCAGAAATTGCAGTAGAAAGTGCAAAATCAGCA
TTA
>tpi_26
CACGAAACAGATGAAGAAATTAACAAAAAAGCGCATGCTATTTTCAAACATGGTATGA
CACCAATTATTTGTGTTGGTGAAACAGATGAAGAGCGTGAAAGTGGTAAAGCTAACGA
TGTTGTAGGTGAGCAAGTTAAGAAAGCTGTTGCAGGTTTATCTGAAGAGCAACTTAAA
TCAGTTGTAATTGCTTATGAACCAATCTGGGCAATCGGAACTGGTAAATCATCAACATC
TGAAGATGCGAATGAAATGTGTGCATTTGTACGTCAAACTATTGCTGACTTATCAAGCA
AAGAAGTATCAGAAGCAACTCGTATTCAATATGGTGGTAGTGTTAAACCTAACAACATT
AAAGAATACATGGCACAAACTGATATTGATGGGGCATTAGTAGGTGGCGCA
>yqil39
GCGTTTAAAGACGTGCCAGCCTATGATTTAGGTGCGACTTTAATAGAACATATTATTAAA
GAGACGGGTTTGAATCCAAGTGAGATTAATGAAGTCATCATCGGTAACGTACTACAAG
CAGGACAAGGACAAAATCCAGCACGAATTGCTGCTATGAAAGGTGGCTTGCCAGAAA
CAGTACCTGCATTTACAGTGAATAAAGTATGTGGTTCTGGGTTAAAGTCGATTCAATTA
GCATATCAATCTATTGTGACTGGTGAAAATGACATCGTGCTAGCTGGCGGTATGGAGAA
TATGTCTCAATCACCAATGCTTGTCAACAACAGTCGCTTTGGTTTTAAAATGGGACATC
AGTCAATGGTTGATAGCATGGTATATGATGGTTTAACAGATGTATTTAATCAATATCATAT
GGGTATTACTGCTGAAAATTTAGTAGAGCAATATGGTATTTCAAGAGAAGAACAAGATA
CATTTGCTGTAAACTCACAACAAAAAGCAGTACGTGCACAGCAA

ST88
>arcc22
TTATTAATCCAACAAGCTAAATCGAACAGTGACACAACGCCGGCAATGCCATTGGATAC
TTGTGGTGCAATGTCACAGGGTATGATAGGCTATTGGTTGGAAACTGAAATCAATCGCA
TTTTAACTGAAATGAATAGTGATAGAACTGTAGGCACAATCGTTACACGTGTGGAAGTA
GATAAAGATGATCCACGATTTGATAACCTAACTAAACCAATTGGTCCTTTTTATACGAAA
GAAGAAGTTGAAGAATTACAAAAAGAACAGCCAGACTCAGTCTTTAAAGAAGATGCA
GGACGTGGTTATAGAAAAGTAGTTGCGTCACCACTACCTCAATCTATACTAGAACACCA
GTTAATTCGAACTTTAGCAGACGGTAAAAATATTGTCATTGCATGCGGTGGTGGCGGTA
TTCCAGTTATAAAAAAAGAAAATACCTATGAAGGTGTTGAAGCG
>aroe1
AATTTTAATTCTTTAGGATTAGATGATACTTATGAAGCTTTAAATATTCCAATTGAAGATT
TTCATTTAATTAAAGAAATTATTTCGAAAAAAGAATTAGATGGCTTTAATATCACAATTC
CTCATAAAGAACGTATCATACCGTATTTAGATCATGTTGATGAACAAGCGATTAATGCAG

GTGCAGTTAACACTGTTTTGATAAAAGATGACAAGTGGATAGGGTATAATACAGATGGT
ATTGGTTATGTTAAAGGATTGCACAGCGTTTATCCAGATTTAGAAAATGCATACATTTTA
ATTTTGGGCGCAGGTGGTGCAAGTAAAGGTATTGCTTATGAATTAGCAAAATTTGTAAA
GCCCAAATTAACTGTTGCGAATAGAACGATGGCTCGTTTTGAATCTTGGAATTTAAATAT
AAACCAAATTTCATTAGCAGATGCTGAAAAGTATTTA
>glpf14
GGTGCTGATTGGATTGTCATCACAGCTGGATGGGGATTAGCGGTTACAATGGGTGTGTT
TGCTGTCGGTCAATTCTCAGGTGCACATTTAAACCCAGCGGTGTCTTTAGCTCTTGCAT
TAGACGGAAGTTTTGATTGGTCATTAGTTCCTGGTTATATTGTTGCTCAAATGTTAGGTG
CAATTGTCGGAGCAACAATTGTATGGTTAATGTACTTGCCACATTGGAAAGCCACAGAA
GAAGCTGGCGCGAAATTAGGTGTTTTCTCTACAGCACCGGCTATTAAGAATTACTTTGC
CAACTTTTTAAGTGAGATTATCGGAACAATGGCATTAACTTTAGGTATTTTATTTATCGGT
GTAAACAAAATTGCCGATGGTTTAAATCCTTTAATTGTCGGAGCATTAATTGTTGCAATC
GGATTAAGTTTAGGCGGTGCTACTGGTTATGCAATCAACCCAGCACGT
>gmk_23
CGAATATTTGAAGATCCAAGTACATCATATAAGTATTCTATTTCAATGACAACACGTCAA
ATGCGTGAAGGTGAAGTTGATGGCGTAGATTACTTTTTTAAAACTAGGGATGCGTTTGA
AGCTTTAATTAAAGATGACCAATTTATAGAATATGCTGAATATGTAGGCAACTATTATGG
TACACCAGTTCAATATGTTAAAGATACAATGGACGAAGGTCATGATGTATTTTTAGAAAT
TGAAGTAGAAGGTGCAAAGCAAGTTAGAAAGAAATTTCCAGATGCGTTATTTATTTTCT
TAGCACCTCCAAGTTTAGATCACTTGAGAGAGCGATTAGTAGGTAGAGGAACAGAATC
CGATGAGAAAATACAAAGTCGTATTAACGAAGCGCGTAAAGAAGTTGAAATGATGAAT
TTA
>pta_12
GCAACACAATTACAAGCAACAGATTATGTTACACCAATCGTGTTAGGTGATGAGACTAA
GGTTCAATCTTTAGCGCAAAAACTTGATCTTGATATTTCTAATATTGAATTAATTAATCCT
GCGACAAGTGAATTGAAAGCTGAATTAGTTCAATCATTTGTTGAACGACGTAAAGGTA
AAGCGACTGAAGAACAAGCACAAGAATTATTAAACAATGTGAACTACTTCGGTACAAT
GCTTGTTTATGCTGGTAAAGCAGATGGTTTAGTTAGTGGTGCAGCACATTCAACAGGAG
ACACTGTGCGTCCAGCTTTACAAATCATCAAAACGAAACCAGGTGTATCAAGAACATC
AGGTATCTTCTTTATGATTAAAGGTGATGAACAATACATCTTTGGTGATTGTGCAATCAA
TCCAGAACTTGATTCACAAGGACTTGCAGAAATTGCAGTAGAAAGTGCAAAATCAGCA
TTA
>tpi_4
CACGAAACAGATGAAGAAATTAACAAAAAAGCGCACGCTATTTTCAAACATGGAATGA
CTCCAATTATATGTGTTGGTGAAACAGACGAAGAGCGTGAAAGTGGTAAAGCTAACGA
TGTTGTAGGTGAGCAAGTTAAGAAAGCTGTTGCAGGTTTATCTGAAGATCAACTTAAAT
CAGTTGTAATTGCTTATGAACCAATCTGGGCAATCGGAACTGGTAAATCATCAACATCT
GAAGATGCAAATGAAATGTGTGCATTTGTACGTCAAACTATTGCTGACTTATCAAGCAA
AGAAGTATCAGAAGCAACTCGTATTCAATATGGTGGTAGTGTTAAACCTAACAACATTA
AAGAATACATGGCACAAACTGATATTGATGGGGCATTAGTAGGTGGCGCA
>yqil31
GCGTTTAAAGACGTGCCAGCCTATGATTTAGGTGCGACTTTAATAGAACATATTATTAAA
GAGACGGGTTTGAATCCAAGTGAGATTGATGAAGTTATCATCGGTAACGTACTACAAG

CAGGACAAGGACAAAATCCAGCACGAATTGCTGCTATGAAAGGTGGCTTGCCAGAAA
CAGTACTTGCATTTACAGTGAATAAAGTATGTGGTTCTGGGTTAAAGTCGATTCAATTA
GCATATCAATCTATTGTGACTGGTGAAAATGACATCGTGCTAGCTGGCGGTATGGAGAA
TATGTCTCAGTCACCAATGCTTGTCAACAACAGTCGCTTCGGTTTTAAAATGGGACATC
AATCAATGGTTGATAGCATGGTATATGATGGTTTAACAGATGTATTTAATCAATATCATAT
GGGTATTACTGCTGAAAATTTAGTGGAGCAATATGGTATTTCAAGAGAAGAACAAGATA
CATTTGCTGTAAACTCACAACAAAAAGCAGTACGTGCACAGCAA

ST239

>arcc2
TTATTAATCCAACAAGCTAAATCGAACAGTGACACAACGCCGGCAATGCCATTGGATAC
TTGTGGTGCAATGTCACAAGGTATGATAGGCTATTGGTTGGAAACTGAAATCAATCGCA
TTTTAACTGAAATGAATAGTGATAGAACTGTAGGCACAATCGTAACACGTGTGGAAGTA
GATAAAGATGATCCACGATTTGATAACCCAACTAAACCAATTGGTCCTTTTTATACGAA
AGAAGAAGTTGAAGAATTACAAAAAGAACAGCCAGGCTCAGTCTTTAAAGAAGATGC
AGGACGTGGTTATAGAAAAGTAGTTGCGTCACCACTACCTCAATCTATACTAGAACACC
AGTTAATTCGAACTTTAGCAGACGGTAAAAATATTGTCATTGCATGCGGTGGTGGCGGT
ATTCCAGTTATAAAAAAAGAAAATACCTATGAAGGTGTTGAAGCG
>aroe3
AATTTTAATTCTTTAGGATTAGATGATACTTATGAAGCTTTAAATATTCCAATTGAAGATT
TTCATTTAATTAAAGAAATTATTTCGAAAAAAGAATTAGAAGGCTTTAATATCACAATTC
CTCATAAAGAACGTATCATACCGTATTTAGATTATGTTGATGAACAAGCGATTAATGCAG
GTGCAGTTAACACTGTTTTGATAAAAGATGGCAAGTGGATAGGGTATAATACAGATGGT
ATTGGTTATGTTAAAGGATTGCACAGCGTTTATCCAGATTTAGAAAATGCATACATTTTA
ATTTTGGGCGCAGGTGGTGCAAGTAAAGGTATTGCTTATGAATTAGCAAAATTTGTAAA
GCCCAAATTAACTGTTGCGAATAGAACGATGGCTCGTTTTGAATCTTGGAATTTAAATAT
AAACCAAATTTCATTAGCAGATGCTGAAAAGTATTTA
>glpf1
GGTGCTGATTGGATTGTCATCACAGCTGGATGGGGATTAGCGGTTACAATGGGTGTGTT
TGCTGTCGGTCAATTCTCAGGTGCACATTTAAACCCAGCGGTGTCTTTAGCTCTTGCAT
TAGACGGAAGTTTTGATTGGTCATTAGTTCCTGGTTATATTGTTGCTCAAATGTTAGGTG
CAATTGTCGGAGCAACAATTGTATGGTTAATGTACTTGCCACATTGGAAAGCGACAGA
AGAAGCTGGCGCGAAATTAGGTGTTTTCTCTACAGCACCGGCTATTAAGAATTACTTTG
CCAACTTTTTAAGTGAGATTATCGGAACAATGGCATTAACTTTAGGTATTTTATTTATCG
GTGTAAACAAAATTGCCGATGGTTTAAATCCTTTAATTGTCGGAGCATTAATTGTTGCA
ATCGGATTAAGTTTAGGCGGTGCTACTGGTTATGCAATCAACCCAGCACGT
>gmk_1
CGAATATTTGAAGATCCAAGTACATCATATAAGTATTCTATTTCAATGACAACACGTCAA
ATGCGTGAAGGTGAAGTTGATGGCGTAGATTACTTTTTTAAAACTAGGGATGCGTTTGA
AGCTTTAATCAAAGATGACCAATTTATAGAATATGCTGAATATGTAGGCAACTATTATGG
TACACCAGTTCAATATGTTAAAGATACAATGGACGAAGGTCATGATGTATTTTTAGAAAT
TGAAGTAGAAGGTGCAAAGCAAGTTAGAAAGAAATTTCCAGATGCGCTATTTATTTTCT
TAGCACCTCCAAGTTTAGAACACTTGAGAGAGCGATTAGTAGGTAGAGGAACAGAATC
TGATGAGAAAATACAAAGTCGTATTAACGAAGCGCGTAAAGAAGTTGAAATGATGAAT

TTA
>pta_4
GCAACACAATTACAAGCAACAGATTATGTTACACCAATCGTGTTAGGTGATGAGACTAA
GGTTCAATCTTTAGCGCAAAAACTTGATCTTGATATTTCTAATATTGAATTAATTAATCCT
GCGACAAGTGAATTGAAAGCTGAATTAGTTCAATCATTTGTTGAACGACGTAAAGGTA
AAGCGACTGAAGAACAAGCACAAGAATTATTAAACAATGTGAACTACTTCGGTACAAT
GCTTGTTTATGCTGGTAAAGCAGATGGTTTAGTTAGTGGTGCAGCACATTCAACAGGCG
ACACTGTGCGTCCAGCTTTACAAATCATCAAAACGAAACCAGGTGTATCAAGAACATC
AGGTATCTTCTTTATGATTAAAGGTGATGAACAATACATCTTTGGTGATTGTGCAATCAA
TCCAGAACTTGATTCACAAGGACTTGCAGAAATTGCAGTAGAAAGTGCAAAATCAGCA
TTA
>tpi_4
CACGAAACAGATGAAGAAATTAACAAAAAAGCGCACGCTATTTTCAAACATGGAATGA
CTCCAATTATATGTGTTGGTGAAACAGACGAAGAGCGTGAAAGTGGTAAAGCTAACGA
TGTTGTAGGTGAGCAAGTTAAGAAAGCTGTTGCAGGTTTATCTGAAGATCAACTTAAAT
CAGTTGTAATTGCTTATGAACCAATCTGGGCAATCGGAACTGGTAAATCATCAACATCT
GAAGATGCAAATGAAATGTGTGCATTTGTACGTCAAACTATTGCTGACTTATCAAGCAA
AGAAGTATCAGAAGCAACTCGTATTCAATATGGTGGTAGTGTTAAACCTAACAACATTA
AAGAATACATGGCACAAACTGATATTGATGGGGCATTAGTAGGTGGCGCA
>yqil3
GCGTTTAAAGACGTGCCAGCCTATGATTTAGGTGCGACTTTAATAGAACATATTATTAAA
GAGACGGGTTTGAATCCAAGTGAGATTGATGAAGTTATCATCGGTAACGTACTACAAG
CAGGACAAGGACAAAATCCAGCACGAATTGCTGCTATGAAAGGTGGCTTGCCAGAAA
CAGTACCTGCATTTACGGTGAATAAAGTATGTGGTTCTGGGTTAAAGTCGATTCAATTA
GCATATCAATCTATTGTGACTGGTGAAAATGACATCGTGCTAGCTGGCGGTATGGAGAA
TATGTCTCAATCACCAATGCTTGTCAACAACAGTCGCTTTGGTTTTAAAATGGGACATC
AATCAATGGTTGATAGCATGGTATATGATGGTTTAACAGATGTATTTAATCAATATCATAT
GGGTATTACTGCTGAAAATTTAGTAGAGCAATATGGTATTTCAAGAGAAGAACAAGATA
CATTTGCTGTAAACTCACAACAAAAAGCAGTACGTGCACAGCAA

ST22
>arcc7
TTATTAATCCAACAAGCTAAATCGAACAGTGACACAACGCCGGCAATGCCATTGGATAC
TTGTGGTGCAATGTCACAGGGTATGATAGGCTATTGGTTGGAAACTGAAATCAATCGCA
TTTTAACTGAAATGAATAGTGATAGAACTGTAGGCACAATCGTTACACGTGTGGAAGTA
GATAAAGATGATCCACGATTCAATAACCCAACCAAACCAATTGGTCCTTTTTATACGAA
AGAAGAAGTTGAAGAATTACAAAAAGAACAGCCAGACTCAGTCTTTAAAGAAGATGC
AGGACGTGGTTATAGAAAAGTTGTTGCGTCACCACTACCTCAATCTATACTAGAACACC
AGTTAATTCGAACTTTAGCAGACGGTAAAAATATTGTCATTGCATGCGGTGGTGGCGGT
ATTCCAGTTATAAAAAAAGAAAATACCTATGAAGGTGTTGAAGCG
>aroe6
AATTTTAATTCTTTAGGATTAGATGATACTTATGAAGCTTTAAATATTCCAATTGAAGATT
TTCATTTAATTAAAGAAATTATTTCGAAAAAAGAATTAGATGGCTTTAATATCACAATTC
CTCATAAAGAGCGTATCATACCGTATTTAGATCATGTTGATGAACAAGCGATTAATGCAG

GTGCAGTTAACACTGTTTTGATAAAAGATGGCAAGTGGATAGGGTATAATACAGATGGT
ATTGGTTATGTTAAAGGATTGCACAGCGTTTATCCAGATTTAGAAAATGCATACATTTTA
ATTTTGGGCGCAGGTGGTGCAAGTAAAGGTATTGCTTATGAATTAGCAAAATTTGTAAA
GCCCAAATTAACTGTTGCGAATAGAACGATGGCTCGTTTTGAATCTTGGAATTTAAATAT
AAACCAAATTTCAATAGCAGATGCTGAAAAGTATTTA
>glpf1
GGTGCTGATTGGATTGTCATCACAGCTGGATGGGGATTAGCGGTTACAATGGGTGTGTT
TGCTGTCGGTCAATTCTCAGGTGCACATTTAAACCCAGCGGTGTCTTTAGCTCTTGCAT
TAGACGGAAGTTTTGATTGGTCATTAGTTCCTGGTTATATTGTTGCTCAAATGTTAGGTG
CAATTGTCGGAGCAACAATTGTATGGTTAATGTACTTGCCACATTGGAAAGCGACAGA
AGAAGCTGGCGCGAAATTAGGTGTTTTCTCTACAGCACCGGCTATTAAGAATTACTTTG
CCAACTTTTTAAGTGAGATTATCGGAACAATGGCATTAACTTTAGGTATTTTATTTATCG
GTGTAAACAAAATTGCCGATGGTTTAAATCCTTTAATTGTCGGAGCATTAATTGTTGCA
ATCGGATTAAGTTTAGGCGGTGCTACTGGTTATGCAATCAACCCAGCACGT
>gmk_5
CGAATATTTGAAGATCCAAGTACATCATATAAGTATTCTATTTCAATGACAACACGTCAA
ATGCGTGAAGGTGAAGTTGATGGCGTAGATTACTTTTTTAAAACTAGGGATGCGTTTGA
AGCTTTAATCAAAGATGACCAATTTATAGAATATGCTGAATATGTAGGCAACTATTATGG
TACACCAGTTCAATATGTTAAAGATACAATGGACGAAGGTCATGATGTATTTTTAGAAAT
TGAAGTAGAAGGTGCAAAGCAAGTTAGAAAGAAATTTCCAGATGCATTATTTATTTTCT
TAGCACCTCCAAGTTTAGATCACTTGAGAGAGCGATTAGTAGGTAGAGGAACAGAATC
CGATGAGAAAATACAAAGTCGTATTAACGAAGCACGTAAAGAAGTCGAAATGATGAAT
TTA
>pta_8
GCAACACAATTACAAGCAACAGATTATGTTACACCAATCGTGTTAGGTGATGAGACTAA
GGTTCAATCTTTAGCGCAAAAACTTAATCTTGATATTTCTAATATTGAATTAATTAATCCT
GCGACAAGTGAATTTAAAGCTGAATTAGTCCAATCATTTGTTGAACGACGTAAAGGTA
AAGCGACTGAAGAACAAGCGCAAGAATTATTAAACAATGTGAACTACTTCGGTACAAT
GCTTGTTTATGCTGGTAAAGCAGATGGTTTAGTTAGTGGTGCAGCACATTCAACAGGAG
ACACTGTGCGTCCAGCTTTACAAATCATCAAAACGAAACCAGGTGTATCAAGAACATC
AGGTATCTTCTTTATGATTAAAGGTGATGAACAATACATCTTTGGTGATTGTGCAATCAA
TCCAGAACTTGATTCACAAGGACTTGCAGAAATTGCAGTAGAAAGTGCAAAATCAGCA
TTA
>tpi_8
CACGAAACAGATAAAGAAATTAACAAAAAAGCGCACGCTATTTTCAAACATGGAATGA
CTCCAATTATATGTGTTGGTGAAACAGACGAAGAGCGTGAAAGTGGTAAAGCTAACGA
TGTTGTAGGTGAGCAAGTTAAGAAAGCTGTTGCAGGTTTATCTGAAAATCAACTAAAA
TCAGTTGTAATTGCTTATGAACCAATCTGGGCAATCGGAACTGGTAAATCATCAACATC
TGAAGATGCGAATGAAATGTGTGCATTTGTACGTCAAACTATTGCTGACTTATCAAGCA
AAGAAGTATCAGAAGCAACTCGTATTCAATATGGTGGTAGTGTTAAACCTAACAACATT
AAAGAATACATGGCACAAACTGATATTGATGGGGCATTAGTAGGTGGCGCA
>yqil6
GCGTTTAAAGACGTGCCAGCCTATGATTTAGGTGCGACTTTAATAGAACATATTATTAAA
GAGACGGGTTTGAATCCAAGTGAGATTGATGAAGTTATCATCGGTAACGTACTACAAG

CAGGACAAGGACAAAATCCAGCACGAATTGCTGCTATGAAAGGTGGCTTACCAGAGA
CAGTACCTGCATTTACAGTGAATAAAGTATGTGGTTCTGGGTTAAAGTCGATTCAATTA
GCATATCAATCTATTGTGACTGGTGAAAATGACATCGTGCTAGCTGGCGGTATGGAGAA
TATGTCTCAATCACCAATGCTTGTCAACAACAGTCGCTTTGGTTTTAAAATGGGACATC
AATCAATGGTTGATAGCATGGTATATGATGGTTTAACAGATGTATTTAATCAATATCATAT
GGGTATTACTGCTGAAAATTTAGTAGAGCAATATGGTATTTCAAGAGAAGAACAAGATA
CATTTGCTGTAAACTCACAACAAAAAGCAGTACGTGCACAGCAA

ST217

>arcc7
TTATTAATCCAACAAGCTAAATCGAACAGTGACACAACGCCGGCAATGCCATTGGATAC
TTGTGGTGCAATGTCACAGGGTATGATAGGCTATTGGTTGGAAACTGAAATCAATCGCA
TTTTAACTGAAATGAATAGTGATAGAACTGTAGGCACAATCGTTACACGTGTGGAAGTA
GATAAAGATGATCCACGATTCAATAACCCAACCAAACCAATTGGTCCTTTTTATACGAA
AGAAGAAGTTGAAGAATTACAAAAAGAACAGCCAGACTCAGTCTTTAAAGAAGATGC
AGGACGTGGTTATAGAAAAGTTGTTGCGTCACCACTACCTCAATCTATACTAGAACACC
AGTTAATTCGAACTTTAGCAGACGGTAAAAATATTGTCATTGCATGCGGTGGTGGCGGT
ATTCCAGTTATAAAAAAAGAAAATACCTATGAAGGTGTTGAAGCG
>aroe6
AATTTTAATTCTTTAGGATTAGATGATACTTATGAAGCTTTAAATATTCCAATTGAAGATT
TTCATTTAATTAAAGAAATTATTTCGAAAAAAGAATTAGATGGCTTTAATATCACAATTC
CTCATAAAGAGCGTATCATACCGTATTTAGATCATGTTGATGAACAAGCGATTAATGCAG
GTGCAGTTAACACTGTTTTGATAAAAGATGGCAAGTGGATAGGGTATAATACAGATGGT
ATTGGTTATGTTAAAGGATTGCACAGCGTTTATCCAGATTTAGAAAATGCATACATTTTA
ATTTTGGGCGCAGGTGGTGCAAGTAAAGGTATTGCTTATGAATTAGCAAAATTTGTAAA
GCCCAAATTAACTGTTGCGAATAGAACGATGGCTCGTTTTGAATCTTGGAATTTAAATAT
AAACCAAATTTCAATAGCAGATGCTGAAAAGTATTTA
>glpf1
GGTGCTGATTGGATTGTCATCACAGCTGGATGGGGATTAGCGGTTACAATGGGTGTGTT
TGCTGTCGGTCAATTCTCAGGTGCACATTTAAACCCAGCGGTGTCTTTAGCTCTTGCAT
TAGACGGAAGTTTTGATTGGTCATTAGTTCCTGGTTATATTGTTGCTCAAATGTTAGGTG
CAATTGTCGGAGCAACAATTGTATGGTTAATGTACTTGCCACATTGGAAAGCGACAGA
AGAAGCTGGCGCGAAATTAGGTGTTTTCTCTACAGCACCGGCTATTAAGAATTACTTTG
CCAACTTTTTAAGTGAGATTATCGGAACAATGGCATTAACTTTAGGTATTTTATTTATCG
GTGTAAACAAAATTGCCGATGGTTTAAATCCTTTAATTGTCGGAGCATTAATTGTTGCA
ATCGGATTAAGTTTAGGCGGTGCTACTGGTTATGCAATCAACCCAGCACGT
>gmk_5
CGAATATTTGAAGATCCAAGTACATCATATAAGTATTCTATTTCAATGACAACACGTCAA
ATGCGTGAAGGTGAAGTTGATGGCGTAGATTACTTTTTTAAAACTAGGGATGCGTTTGA
AGCTTTAATCAAAGATGACCAATTTATAGAATATGCTGAATATGTAGGCAACTATTATGG
TACACCAGTTCAATATGTTAAAGATACAATGGACGAAGGTCATGATGTATTTTTAGAAAT
TGAAGTAGAAGGTGCAAAGCAAGTTAGAAAGAAATTTCCAGATGCATTATTTATTTTCT
TAGCACCTCCAAGTTTAGATCACTTGAGAGAGCGATTAGTAGGTAGAGGAACAGAATC

CGATGAGAAAATACAAAGTCGTATTAACGAAGCACGTAAAGAAGTCGAAATGATGAAT
TTA
>pta_8
GCAACACAATTACAAGCAACAGATTATGTTACACCAATCGTGTTAGGTGATGAGACTAA
GGTTCAATCTTTAGCGCAAAAACTTAATCTTGATATTTCTAATATTGAATTAATTAATCCT
GCGACAAGTGAATTTAAAGCTGAATTAGTCCAATCATTTGTTGAACGACGTAAAGGTA
AAGCGACTGAAGAACAAGCGCAAGAATTATTAAACAATGTGAACTACTTCGGTACAAT
GCTTGTTTATGCTGGTAAAGCAGATGGTTTAGTTAGTGGTGCAGCACATTCAACAGGAG
ACACTGTGCGTCCAGCTTTACAAATCATCAAAACGAAACCAGGTGTATCAAGAACATC
AGGTATCTTCTTTATGATTAAAGGTGATGAACAATACATCTTTGGTGATTGTGCAATCAA
TCCAGAACTTGATTCACAAGGACTTGCAGAAATTGCAGTAGAAAGTGCAAAATCAGCA
TTA
>tpi_5
CACGAAACAGATGAAGAAATTAACAAAAAAGCGCACGCTATTTTCAAACATGGAATGA
CTCCAATTATATGTGTTGGTGAAACAGACGAAGAGCGTGAAAGTGGTAAAGCTAACGA
TGTTGTAGGTGAGCAAGTTAAGAAAGCTGTTGCAGGTTTATCTGAAAATCAACTAAAA
TCAGTTGTAATTGCTTATGAACCAATCTGGGCAATCGGAACTGGTAAATCATCAACATC
TGAAGATGCGAATGAAATGTGTGCATTTGTACGTCAAACTATTGCTGACTTATCAAGCA
AAGAAGTATCAGAAGCAACTCGTATTCAATATGGTGGTAGTGTTAAACCTAACAACATT
AAAGAATACATGGCACAAACTGATATTGATGGGGCATTAGTAGGTGGCGCA
>yqil6
GCGTTTAAAGACGTGCCAGCCTATGATTTAGGTGCGACTTTAATAGAACATATTATTAAA
GAGACGGGTTTGAATCCAAGTGAGATTGATGAAGTTATCATCGGTAACGTACTACAAG
CAGGACAAGGACAAAATCCAGCACGAATTGCTGCTATGAAAGGTGGCTTACCAGAGA
CAGTACCTGCATTTACAGTGAATAAAGTATGTGGTTCTGGGTTAAAGTCGATTCAATTA
GCATATCAATCTATTGTGACTGGTGAAAATGACATCGTGCTAGCTGGCGGTATGGAGAA
TATGTCTCAATCACCAATGCTTGTCAACAACAGTCGCTTTGGTTTTAAAATGGGACATC
AATCAATGGTTGATAGCATGGTATATGATGGTTTAACAGATGTATTTAATCAATATCATAT
GGGTATTACTGCTGAAAATTTAGTAGAGCAATATGGTATTTCAAGAGAAGAACAAGATA
CATTTGCTGTAAACTCACAACAAAAAGCAGTACGTGCACAGCAA

ST121

>arcc6
TTATTAATCCAACAAGCTAAATCGAACAGTGACACAACGCCGGCAATGCCATTGGATAC
TTGTGGTGCAATGTCACAGGGTATGATAGGCTATTGGTTGGAAACTGAAATCAATCGCA
TTTTAACTGAAATGAATAGTGATAGAACTGTAGGCACAATCGTTACACGTGTGGAAGTA
GATAAAAATGATCCACGATTTGATAACCCAACTAAACCAATTGGTCCTTTTTATACGAA
AGAAGAAGTTGAAGAATTACAAAAAGAACAGCCAGACTCAGTCTTTAAAGAAGATGC
AGGACGTGGTTATAGAAAAGTAGTTGCGTCACCACTACCTCAATCTATACTAGAACACC
AGTTAATTCGAACTTTAGCAGACGGTAAAAATATTGTCATTGCATGCGGTGGTGGCGGT
ATTCCAGTTATAAAAAAAGAAAATACCTATGAAGGTGTTGAAGCG
>aroe5
AATTTTAATTCTTTAGGATTAGATGATACTTATGAAGCTTTAAATATTCCAATTGAAGATT
TTCATTTAATTAAAGAAATTATTTCAAAAAAAGAATTAGATGGCTTTAATATCACAATTC

CTCATAAAGAGCGTATCATACCGTATTTAGATCATGTTGATGAACAAGCGATTAATGCAG
GTGCAGTTAACACTGTTTTGATAAAAGATGGCAAGTGGATAGGGTATAATACAGATGGT
ATTGGTTATGTAAAGGGATTGCACAGCGTTTATCCAGATTTAGAAAATGCATACATTTTA
ATTTTGGGCGCAGGTGGTGCAAGTAAAGGTATTGCTTATGAATTAGCAAAATTTGTAAA
GCCCAAATTAACTGTTGCGAATAGAACGATGGCTCGTTTTGAATCTTGGAATTTAAATAT
AAACCAAATTTCATTGGAAGATGCTGAAAAGTATTTA
>glpf6
GGTGCTGATTGGATTGTCATCACAGCTGGATGGGGATTAGCGGTTACAATGGGTGTGTA
TGCTGTCGGTCAATTCTCAGGTGCACATTTAAACCCAGCGGTGTCTTTAGCTCTTGCAT
TAGACGGAAGTTTTGATTGGTCATTAGTTCCTGGTTATATTGTTGCTCAAATGTTAGGTG
CAATTGTCGGAGCAACAATTGTATGGTTAATGTACTTGCCACATTGGAAAGCGACAGA
AGAAGCTGGCGCGAAATTAGGTGTTTTCTCTACAGCACCGGCTATTAAGAATTACTTTG
CCAACTTTTTAAGTGAGATTATCGGAACAATGGCATTAACTTTAGGTATTTTATTTATCG
GTGTAAACAAAATTGCCGATGGTTTAAATCCTTTAATTGTCGGAGCATTAATTGTTGCA
ATCGGATTAAGTTTAGGCGGTGCTACTGGTTATGCAATCAACCCAGCACGT
>gmk_2
CGAATATTTGAAGATCCAAGTACATCATATAAGTATTCTATTTCAATGACAACACGTCAA
ATGCGTGAAGGTGAAGTTGATGGCGTAGATTACTTTTTAAAACTAGGGATGCGTTTGA
AGCTTTAATTAAAGATGACCAATTTATAGAATATGCTGAATATGTAGGCAACTATTATGG
TACACCAGTTCAATATGTTAAAGATACAATGGACGAAGGTCATGATGTATTTTTAGAAAT
TGAAGTAGAAGGTGCAAAGCAAGTTAGAAAGAAATTTCCAGATGCGTTATTTATTTTCT
TAGCACCTCCAAGTTTAGATCACTTGAGAGAGCGATTAGTAGGTAGAGGAACAGAATC
TGATGAGAAAATACAAAGTCGTATTAACGAAGCACGTAAAGAAGTCGAAATGATGAAT
TTA
>pta_7
GCAACACAATTACAAGCAACAGATTATGTTACACCAATCGTGTTAGGTGATGAGACTAA
GGTTCAATCTTTAGCGCAAAAACTTAATCTTGATATTTCTAATATTGAATTAATTAATCCT
GCGACAAGTGAATTGAAAGCTGAATTAGTTCAATCATTTGTTGAACGACGTAAAGGTA
AAGCGACTGAAGAACAAGCACAAGAATTATTAAACAATGTGAACTACTTCGGTACAAT
GCTTGTTTATGCTGGTAAAGCAGATGGTCTAGTTAGTGGTGCAGCACATTCAACAGGCG
ACACTGTGCGTCCAGCTTTACAAATCATCAAAACGAAACCAGGTGTATCAAGAACATC
AGGTATCTTCTTTATGATTAAAGGTGATGAACAATACATCTTTGGTGATTGTGCAATCAA
TCCAGAACTTGATTCACAAGGACTTGCAGAAATTGCAGTAGAAAGTGCAAAATCAGCA
TTA
>tpi_14
CACGAAACAGATGAAGAAATTAACAAAAAAGCGCATGCTATTTTCAAACATGGAATGA
CTCCAATTATTTGTGTTGGTGAAACAGACGAAGAGCGTGAAAGTGGTAAAGCTAACGA
TGTTGTAGGTGAGCAAGTTAAGAAAGCTGTTGCAGGTTTATCTGAAGATCAACTTAAAT
CAGTTATAATTGCTTATGAACCAATTTGGGCAATCGGAACTGGTAAATCATCAACATCTG
AAGATGCGAATGAAATGTGTGCATTTGTACGTCAAACTATTGCTGACTTATCAAGCAAA
GAAGTATCAGAAGCAACTCGTATTCAATATGGTGGTAGTGTTAAACCTAACAACATTAA
AGAATACATGGCACAAACTGATATTGATGGGGCATTAGTAGGTGGCGCA
>yqil5
GCGTTTAAAGACGTGCCAGCCTATGATTTAGGTGCGACTTTAATAGAACATATTATTAAA

GAGACGGGTTTGAATCCAAGTGAGATTAATGAAGTCATCATCGGTAACGTACTACAAG
CAGGACAAGGACAAAATCCAGCACGAATTGCTGCTATGAAAGGTGGCTTGCCAGAAA
CAGTACCTGCATTTACAGTGAATAAAGTATGTGGTTCTGGGTTAAAGTCGATTCAATTA
GCATATCAATCTATTGTGACTGGTGAAAATGACATCGTGCTAGCTGGCGGTATGGAGAA
TATGTCTCAATCACCAATGCTTGTCAACAACAGTCGCTTTGGTTTTAAAATGGGACATC
AATCAATGGTTGATAGCATGGTATATGATGGTTTAACAGATGTATTTAATCAATATCATAT
GGGTATTACTGCTGAAAATTTAGTAGAGCAATATGGTATTTCAAGAGAAGAACAAGATA
CATTTGCTGTAAACTCACAACAAAAAGCAGTACGTGCACAACAA

ST9

>arcc3
TTATTAATCCAACAAGCTAAATCGAACAGTGACACAACGCCGGCAATGCCATTGGATAC
TTGTGGTGCAATGTCACAGGGTATGATAGGCTATTGGTTGGAAACTGAAATCAATCGCA
TTTTAACTGAAATGAATAGTGATAGAACTGTAGGCACAATCGTTACACGTGTGGAAGTA
GATAAAGATGATCCACGATTTGATAACCCAACTAAACCAATTGGTCCTTTTTATACGAA
AGAAGAAGTTGAAGAATTACAAAAAGAACAGCCAGACTCAGTCTTTAAAGAAGATGC
AGGACGTGGTTATAGAAAAGTAGTTGCGTCACCACTACCTCAATCTATACTAGAACACC
AGTTAATTCGAACTTTAGCAGACGGTAAAAATATTGTCATTGCATGCGGTGGTGGCGGT
ATTCCAGTTATAAAAAAAGAAAATACCTATGAAGGTGTTGAAGCG
>aroe3
AATTTTAATTCTTTAGGATTAGATGATACTTATGAAGCTTTAAATATTCCAATTGAAGATT
TTCATTTAATTAAAGAAATTATTTCGAAAAAAGAATTAGAAGGCTTTAATATCACAATTC
CTCATAAAGAACGTATCATACCGTATTTAGATTATGTTGATGAACAAGCGATTAATGCAG
GTGCAGTTAACACTGTTTTGATAAAAGATGGCAAGTGGATAGGGTATAATACAGATGGT
ATTGGTTATGTTAAAGGATTGCACAGCGTTTATCCAGATTTAGAAAATGCATACATTTTA
ATTTTGGGCGCAGGTGGTGCAAGTAAAGGTATTGCTTATGAATTAGCAAAATTTGTAAA
GCCCAAATTAACTGTTGCGAATAGAACGATGGCTCGTTTTGAATCTTGGAATTTAAATAT
AAACCAAATTTCATTAGCAGATGCTGAAAAGTATTTA
>glpf1
GGTGCTGATTGGATTGTCATCACAGCTGGATGGGGATTAGCGGTTACAATGGGTGTGTT
TGCTGTCGGTCAATTCTCAGGTGCACATTTAAACCCAGCGGTGTCTTTAGCTCTTGCAT
TAGACGGAAGTTTTGATTGGTCATTAGTTCCTGGTTATATTGTTGCTCAAATGTTAGGTG
CAATTGTCGGAGCAACAATTGTATGGTTAATGTACTTGCCACATTGGAAAGCGACAGA
AGAAGCTGGCGCGAAATTAGGTGTTTTCTCTACAGCACCGGCTATTAAGAATTACTTTG
CCAACTTTTTAAGTGAGATTATCGGAACAATGGCATTAACTTTAGGTATTTTATTTATCG
GTGTAAACAAAATTGCCGATGGTTTAAATCCTTTAATTGTCGGAGCATTAATTGTTGCA
ATCGGATTAAGTTTAGGCGGTGCTACTGGTTATGCAATCAACCCAGCACGT
>gmk_1
CGAATATTTGAAGATCCAAGTACATCATATAAGTATTCTATTTCAATGACAACACGTCAA
ATGCGTGAAGGTGAAGTTGATGGCGTAGATTACTTTTTTAAAACTAGGGATGCGTTTGA
AGCTTTAATCAAAGATGACCAATTTATAGAATATGCTGAATATGTAGGCAACTATTATGG
TACACCAGTTCAATATGTTAAAGATACAATGGACGAAGGTCATGATGTATTTTTAGAAAT
TGAAGTAGAAGGTGCAAAGCAAGTTAGAAAGAAATTTCCAGATGCGCTATTTATTTTCT
TAGCACCTCCAAGTTTAGAACACTTGAGAGAGCGATTAGTAGGTAGAGGAACAGAATC

TGATGAGAAAATACAAAGTCGTATTAACGAAGCGCGTAAAGAAGTTGAAATGATGAAT
TTA
>pta_1
GCAACACAATTACAAGCAACAGATTATGTTACACCAATCGTGTTAGGTGATGAGACTAA
GGTTCAATCTTTAGCGCAAAAACTTGATCTTGATATTTCTAATATTGAATTAATTAATCCT
GCGACAAGTGAATTGAAAGCTGAATTAGTTCAATCATTTGTTGAACGACGTAAAGGTA
AAGCGACTGAAGAACAAGCACAAGAATTATTAAACAATGTGAACTACTTCGGTACAAT
GCTTGTTTATGCTGGTAAAGCAGATGGTTTAGTTAGTGGTGCAGCACATTCAACAGGAG
ACACTGTGCGTCCAGCTTTACAAATCATCAAAACGAAACCAGGTGTATCAAGAACATC
AGGTATCTTCTTTATGATTAAAGGTGATGTACAATACATCTTTGGTGATTGTGCAATCAA
TCCAGAACTTGATTCACAAGGACTTGCAGAAATTGCAGTAGAAAGTGCAAAATCAGCA
TTA
>tpi_1
CACGAAACAGATGAAGAAATTAACAAAAAAGCGCACGCTATTTTCAAACATGGAATGA
CTCCAATTATTTGTGTTGGTGAAACAGACGAAGAGCGTGAAAGTGGTAAAGCTAACGA
TGTTGTAGGTGAGCAAGTTAAGAAAGCTGTTGCAGGTTTATCTGAAGATCAACTTAAAT
CAGTTGTAATTGCTTATGAGCCAATCTGGGCAATCGGAACTGGTAAATCATCAACATCT
GAAGATGCAAATGAAATGTGTGCATTTGTACGTCAAACTATTGCTGACTTATCAAGCAA
AGAAGTATCAGAAGCAACTCGTATTCAATATGGTGGTAGTGTTAAACCTAACAACATTA
AAGAATACATGGCACAAACTGATATTGATGGGGCATTAGTAGGTGGCGCA
>yqil10
GCGTTTAAAGACGTGCCAGCCTATGATTTAGGTGCGACTTTAATAGAACATATTATTAAA
GAGACGGGTTTGAATCCAAGTGAGATTGATGAAGTTATCATCGGTAACGTACTACAAG
CAGGACAAGGACAAAATCCAGCACGAATTGCTGCTATGAAAGGTGGCTTGCCAGAAA
CAGTACCTGCATTTACAGTGAATAAAGTATGTGGTTCTGGGTTAAAGTCGATTCAATTA
GCATATCAATCTATTGTGACTGGTGAAAATGACATCGTGCTAGCTGGCGGTATGGAGAA
TATGTCTCAGTCACCAATGCTTGTCAACAACAGTCGCTTCGGTTTTAAAATGGGACATC
AATCAATGGTTGATAGCATGGTATATGATGGTTTAACAGATGTATTTAATCAATATCATAT
GGGTATTACTGCTGAAAATTTAGTAGAGCAATATGGTATTTCAAGAGAAGAACAAGATA
CATTTGCTGTAAACTCACAACATAAAGCAGTACGTGCACAGCAA

ST2590
>arcc1
TTATTAATCCAACAAGCTAAATCGAACAGTGACACAACGCCGGCAATGCCATTGGATAC
TTGTGGTGCAATGTCACAGGGTATGATAGGCTATTGGTTGGAAACTGAAATCAATCGCA
TTTTAACTGAAATGAATAGTGATAGAACTGTAGGCACAATCGTTACACGTGTGGAAGTA
GATAAAGATGATCCACGATTCAATAACCCAACCAAACCAATTGGTCCTTTTTATACGAA
AGAAGAAGTTGAAGAATTACAAAAAGAACAGCCAGACTCAGTCTTTAAAGAAGATGC
AGGACGTGGTTATAGAAAAGTAGTTGCGTCACCACTACCTCAATCTATACTAGAACACC
AGTTAATTCGAACTTTAGCAGACGGTAAAAATATTGTCATTGCATGCGGTGGTGGCGGT
ATTCCAGTTATAAAAAAAGAAAATACCTATGAAGGTGTTGAAGCG
>aroe4
AATTTTAATTCTTTAGGATTAGATGATACTTATGAAGCTTTAAATATTCCAATTGAAGATT
TTCATTTAATTAAAGAAATTATTTCGAAAAAAGAATTAGATGGCTTTAATATCACAATTC

CTCATAAAGAACGTATCATACCGTATTTAGATTATGTTGATGAACAAGCGATTAATGCAG
GTGCAGTTAACACTGTTTTGATAAAAGATGGCAAGTGGATAGGGTATAATACAGATGGT
ATTGGTTATGTTAAAGGATTGCACAGCGTTTATCCAGATTTAGAAAATGCATACATTTTA
ATTTTGGGCGCAGGTGGTGCAAGTAAAGGTATTGCTTATGAATTAGCAAAATTTGTAAA
GCCCAAATTAACTGTTGCGAATAGAACGATGGCTCGTTTTGAATCTTGGAATTTAAATAT
AAACCAAATTTCATTGGCAGATGCTGAAAAGTATTTA
>glpf1
GGTGCTGATTGGATTGTCATCACAGCTGGATGGGGATTAGCGGTTACAATGGGTGTGTT
TGCTGTCGGTCAATTCTCAGGTGCACATTTAAACCCAGCGGTGTCTTTAGCTCTTGCAT
TAGACGGAAGTTTTGATTGGTCATTAGTTCCTGGTTATATTGTTGCTCAAATGTTAGGTG
CAATTGTCGGAGCAACAATTGTATGGTTAATGTACTTGCCACATTGGAAAGCGACAGA
AGAAGCTGGCGCGAAATTAGGTGTTTTCTCTACAGCACCGGCTATTAAGAATTACTTTG
CCAACTTTTTAAGTGAGATTATCGGAACAATGGCATTAACTTTAGGTATTTTATTTATCG
GTGTAAACAAAATTGCCGATGGTTTAAATCCTTTAATTGTCGGAGCATTAATTGTTGCA
ATCGGATTAAGTTTAGGCGGTGCTACTGGTTATGCAATCAACCCAGCACGT
>gmk_4
CGAATATTTGAAGATCCAAGTACATCATATAAGTATTCTATTTCAATGACAACACGTCAA
ATGCGTGAAGGTGAAGTTGATGGCGTAGATTACTTTTTTAAAACTAGGGATGCGTTTGA
AGCTTTAATTAAAGATGACCAATTTATAGAATATGCTGAATATGTAGGCAACTATTATGG
TACACCAGTTCAATATGTTAAAGATACAATGGACGAAGGTCATGATGTATTTTTAGAAAT
TGAAGTAGAAGGTGCAAAGCAAGTTAGAAAGAAATTTCCAGATGCGTTATTTATTTTCT
TAGCACCTCCAAGTTTAGATCACTTGAGAGAGCGATTAGTAGGTAGAGGAACAGAATC
CAATGAGAAAATACAAAGTCGTATTAACGAAGCGCGTAAAGAAGTTGAAATGATGAAT
TTA
>pta_12
GCAACACAATTACAAGCAACAGATTATGTTACACCAATCGTGTTAGGTGATGAGACTAA
GGTTCAATCTTTAGCGCAAAAACTTGATCTTGATATTTCTAATATTGAATTAATTAATCCT
GCGACAAGTGAATTGAAAGCTGAATTAGTTCAATCATTTGTTGAACGACGTAAAGGTA
AAGCGACTGAAGAACAAGCACAAGAATTATTAAACAATGTGAACTACTTCGGTACAAT
GCTTGTTTATGCTGGTAAAGCAGATGGTTTAGTTAGTGGTGCAGCACATTCAACAGGAG
ACACTGTGCGTCCAGCTTTACAAATCATCAAAACGAAACCAGGTGTATCAAGAACATC
AGGTATCTTCTTTATGATTAAAGGTGATGAACAATACATCTTTGGTGATTGTGCAATCAA
TCCAGAACTTGATTCACAAGGACTTGCAGAAATTGCAGTAGAAAGTGCAAAATCAGCA
TTA
>tpi_1
CACGAAACAGATGAAGAAATTAACAAAAAAGCGCACGCTATTTTCAAACATGGAATGA
CTCCAATTATTTGTGTTGGTGAAACAGACGAAGAGCGTGAAAGTGGTAAAGCTAACGA
TGTTGTAGGTGAGCAAGTTAAGAAAGCTGTTGCAGGTTTATCTGAAGATCAACTTAAAT
CAGTTGTAATTGCTTATGAGCCAATCTGGGCAATCGGAACTGGTAAATCATCAACATCT
GAAGATGCAAATGAAATGTGTGCATTTGTACGTCAAACTATTGCTGACTTATCAAGCAA
AGAAGTATCAGAAGCAACTCGTATTCAATATGGTGGTAGTGTTAAACCTAACAACATTA
AAGAATACATGGCACAAACTGATATTGATGGGGCATTAGTAGGTGGCGCA
>yqil293
GCGTTTAAAGACGTGCCAGCCTATGATTTAGGTGCGACTTTAATAAAACATATTATTAAA

GAGACGGGTTTGAATCCAAGTGAGATTGATGAAGTTATCATCGGTAACGTACTACAAG
CAGGACAAGGACAAAATCCAGCACGAATTGCTGCTATGAAAGGTGGCTTGCCAGAAA
CAGTACCTGCATTTACAGTGAATAAAGTATGTGGTTCTGGGTTAAAGTCGATTCAATTA
GCATATCAATCTATTGTGACTGGTGAAAATGACATCGTGCTAGCTGGCGGTATGGAGAA
TATGTCTCAGTCACCAATGCTTGTCAACAACAGTCGCTTCGGTTTTAAAATGGGACATC
AATCAATGGTTGATAGCATGGTATATGATGGTTTAACAGATGTATTTAATCAATATCATAT
GGGTATTACTGCTGAAAATTTAGTAGAGCAATATGGTATTTCAAGAGAAGAACAAGATA
CATTTGCTGTAAACTCACAACATAAAGCAGTACGTGCACAGCAA

ST105
>arcc1
TTATTAATCCAACAAGCTAAATCGAACAGTGACACAACGCCGGCAATGCCATTGGATAC
TTGTGGTGCAATGTCACAGGGTATGATAGGCTATTGGTTGGAAACTGAAATCAATCGCA
TTTTAACTGAAATGAATAGTGATAGAACTGTAGGCACAATCGTTACACGTGTGGAAGTA
GATAAAGATGATCCACGATTCAATAACCCAACCAAACCAATTGGTCCTTTTTATACGAA
AGAAGAAGTTGAAGAATTACAAAAAGAACAGCCAGACTCAGTCTTTAAAGAAGATGC
AGGACGTGGTTATAGAAAAGTAGTTGCGTCACCACTACCTCAATCTATACTAGAACACC
AGTTAATTCGAACTTTAGCAGACGGTAAAAATATTGTCATTGCATGCGGTGGTGGCGGT
ATTCCAGTTATAAAAAAAGAAAATACCTATGAAGGTGTTGAAGCG
>aroe4
AATTTAATTCTTTAGGATTAGATGATACTTATGAAGCTTTAAATATTCCAATTGAAGATT
TTCATTTAATTAAAGAAATTATTTCGAAAAAAGAATTAGATGGCTTTAATATCACAATTC
CTCATAAAGAACGTATCATACCGTATTTAGATTATGTTGATGAACAAGCGATTAATGCAG
GTGCAGTTAACACTGTTTTGATAAAAGATGGCAAGTGGATAGGGTATAATACAGATGGT
ATTGGTTATGTTAAAGGATTGCACAGCGTTTATCCAGATTTAGAAAATGCATACATTTTA
ATTTTGGGCGCAGGTGGTGCAAGTAAAGGTATTGCTTATGAATTAGCAAAATTTGTAAA
GCCCAAATTAACTGTTGCGAATAGAACGATGGCTCGTTTTGAATCTTGGAATTTAAATAT
AAACCAAATTTCATTGGCAGATGCTGAAAAGTATTTA
>glpf1
GGTGCTGATTGGATTGTCATCACAGCTGGATGGGGATTAGCGGTTACAATGGGTGTGTT
TGCTGTCGGTCAATTCTCAGGTGCACATTTAAACCCAGCGGTGTCTTTAGCTCTTGCAT
TAGACGGAAGTTTTGATTGGTCATTAGTTCCTGGTTATATTGTTGCTCAAATGTTAGGTG
CAATTGTCGGAGCAACAATTGTATGGTTAATGTACTTGCCACATTGGAAAGCGACAGA
AGAAGCTGGCGCGAAATTAGGTGTTTTCTCTACAGCACCGGCTATTAAGAATTACTTTG
CCAACTTTTTAAGTGAGATTATCGGAACAATGGCATTAACTTTAGGTATTTTATTTATCG
GTGTAAACAAAATTGCCGATGGTTTAAATCCTTTAATTGTCGGAGCATTAATTGTTGCA
ATCGGATTAAGTTTAGGCGGTGCTACTGGTTATGCAATCAACCCAGCACGT
>gmk_4
CGAATATTTGAAGATCCAAGTACATCATATAAGTATTCTATTTCAATGACAACACGTCAA
ATGCGTGAAGGTGAAGTTGATGGCGTAGATTACTTTTTTAAAACTAGGGATGCGTTTGA
AGCTTTAATTAAAGATGACCAATTTATAGAATATGCTGAATATGTAGGCAACTATTATGG
TACACCAGTTCAATATGTTAAAGATACAATGGACGAAGGTCATGATGTATTTTTAGAAAT
TGAAGTAGAAGGTGCAAAGCAAGTTAGAAAGAAATTTCCAGATGCGTTATTTATTTTCT

TAGCACCTCCAAGTTTAGATCACTTGAGAGAGCGATTAGTAGGTAGAGGAACAGAATC
CAATGAGAAAATACAAAGTCGTATTAACGAAGCGCGTAAAGAAGTTGAAATGATGAAT
TTA
>pta_12
GCAACACAATTACAAGCAACAGATTATGTTACACCAATCGTGTTAGGTGATGAGACTAA
GGTTCAATCTTTAGCGCAAAAACTTGATCTTGATATTTCTAATATTGAATTAATTAATCCT
GCGACAAGTGAATTGAAAGCTGAATTAGTTCAATCATTTGTTGAACGACGTAAAGGTA
AAGCGACTGAAGAACAAGCACAAGAATTATTAAACAATGTGAACTACTTCGGTACAAT
GCTTGTTTATGCTGGTAAAGCAGATGGTTTAGTTAGTGGTGCAGCACATTCAACAGGAG
ACACTGTGCGTCCAGCTTTACAAATCATCAAAACGAAACCAGGTGTATCAAGAACATC
AGGTATCTTCTTTATGATTAAAGGTGATGAACAATACATCTTTGGTGATTGTGCAATCAA
TCCAGAACTTGATTCACAAGGACTTGCAGAAATTGCAGTAGAAAGTGCAAAATCAGCA
TTA
>tpi_1
CACGAAACAGATGAAGAAATTAACAAAAAAGCGCACGCTATTTTCAAACATGGAATGA
CTCCAATTATTTGTGTTGGTGAAACAGACGAAGAGCGTGAAAGTGGTAAAGCTAACGA
TGTTGTAGGTGAGCAAGTTAAGAAAGCTGTTGCAGGTTTATCTGAAGATCAACTTAAAT
CAGTTGTAATTGCTTATGAGCCAATCTGGGCAATCGGAACTGGTAAATCATCAACATCT
GAAGATGCAAATGAAATGTGTGCATTTGTACGTCAAACTATTGCTGACTTATCAAGCAA
AGAAGTATCAGAAGCAACTCGTATTCAATATGGTGGTAGTGTTAAACCTAACAACATTA
AAGAATACATGGCACAAACTGATATTGATGGGGCATTAGTAGGTGGCGCA
>yqil28
GCGTTTAAAGACGTGCCAGCCTATGATTTAGGTGCGACTTTAATAGAACATATTATTAAA
GAGACGGGTTTGAATCCAAGTGAGATTGATGAAGTTATCATCGGTAACGTACTACAAG
CAGGACAAGGACAAAATCCAGCACGAATTGCTGCTATGAAAGGTGGCTTGCCAGAAA
AAGTACCTGCATTTACAGTGAATAAAGTATGTGGTTCTGGGTTAAAGTCGATTCAATTA
GCATATCAATCTATTGTGACTGGTGAAAATGACATCGTGCTAGCTGGCGGTATGGAGAA
TATGTCTCAGTCACCAATGCTTGTCAACAACAGTCGCTTCGGTTTTAAAATGGGACATC
AATCAATGGTTGATAGCATGGTATATGATGGTTTAACAGATGTATTTAATCAATATCATAT
GGGTATTACTGCTGAAAATTTAGTAGAGCAATATGGTATTTCAAGAGAAGAACAAGATA
CATTTGCTGTAAACTCACAACATAAAGCAGTACGTGCACAGCAA

ST398
>arcc3
TTATTAATCCAACAAGCTAAATCGAACAGTGACACAACGCCGGCAATGCCATTGGATAC
TTGTGGTGCAATGTCACAGGGTATGATAGGCTATTGGTTGGAAACTGAAATCAATCGCA
TTTTAACTGAAATGAATAGTGATAGAACTGTAGGCACAATCGTTACACGTGTGGAAGTA
GATAAAGATGATCCACGATTTGATAACCCAACTAAACCAATTGGTCCTTTTTATACGAA
AGAAGAAGTTGAAGAATTACAAAAAGAACAGCCAGACTCAGTCTTTAAAGAAGATGC
AGGACGTGGTTATAGAAAAGTAGTTGCGTCACCACTACCTCAATCTATACTAGAACACC
AGTTAATTCGAACTTTAGCAGACGGTAAAAATATTGTCATTGCATGCGGTGGTGGCGGT
ATTCCAGTTATAAAAAAAGAAAATACCTATGAAGGTGTTGAAGCG
>aroe35
AATTTTAATTCTTTGGGATTAGATGATAGTTATGAAGCTTTAAATATTCCAATTGAAGATT

TTCATTTAATTAAAGAAATTATTTCAAAAAAAGAATTAGATGGCTTTAATATCACAATTC
CTCATAAAGAGCGTATCATACCGTATTTAGATCATGTTGATGAACAAGCGATTAATGCAG
GTGCAGTTAATACTGTTTTGATAAAAGATGGCAAGTGGATAGGGTATAATACAGATGGT
ATTGGTTATGTAAAAGGATTGCACAGCGTTTATCCAGATTTAGAAAATGCATACATTTTA
ATTTTGGGAGCAGGTGGTGCAAGTAAAGGTATTGCTTATGAATTAGCAAAATTTGTAAA
GCCCAAATTAACTGTTGCGAATAGAACGTTGGCTCGTTTTGAATCTTGGAATTTAAATAT
AAATCAAATTTCATTGGCAGATGCTGAAAAGTATTTA
>glpf19
GGTGCTGATTGGATTGTCATCACAGCTGGATGGGGATTAGCGGTTACAATGGGTGTGTA
TGCTGTCGGTCAATTCTCAGGTGCACATTTAAACCCAGCGGTGTCTTTAGCTCTTGCAT
TAGACGGAAGTTTTGATTGGTCATTAGTTCCTGGTTATATTGTTGCTCAAATGTTAGGTG
CAATTGTCGGAGCAACAATTGTATGGTTAATGTACTTGCCACATTGGAAAGCGACAGA
AGAAGCTGGCGCGAAATTAGGTGTTTTCTCTACAGCACCAGCTATTAAGAATTACTTTG
CCAACTTTTTAAGTGAGATTATCGGAACAATGGCATTAACTTTAGGTATTTTATTTATCG
GTGTAAACAAAATTGCCGATGGTTTAAATCCTTTAATTGTCGGAGCATTAATTGTTGCA
ATCGGATTAAGTTTAGGCGGTGCTACTGGTTATGCAATCAACCCAGCACGT
>gmk_2
CGAATATTTGAAGATCCAAGTACATCATATAAGTATTCTATTTCAATGACAACACGTCAA
ATGCGTGAAGGTGAAGTTGATGGCGTAGATTACTTTTTAAAACTAGGGATGCGTTTGA
AGCTTTAATTAAAGATGACCAATTTATAGAATATGCTGAATATGTAGGCAACTATTATGG
TACACCAGTTCAATATGTTAAAGATACAATGGACGAAGGTCATGATGTATTTTTAGAAAT
TGAAGTAGAAGGTGCAAAGCAAGTTAGAAAGAAATTTCCAGATGCGTTATTTATTTTCT
TAGCACCTCCAAGTTTAGATCACTTGAGAGAGCGATTAGTAGGTAGAGGAACAGAATC
TGATGAGAAAATACAAAGTCGTATTAACGAAGCACGTAAAGAAGTCGAAATGATGAAT
TTA
>pta_20
GCAACACAATTACAAGCAACAGATTATGTTACACCAATCGTGTTAGGTGATGAGACTAA
GGTTCAATCTTTAGCGCAAAAACTTAATCTTGATATTTCTAATATTGAATTAATTAATCCT
GCGACAAGTGAATTGAAAGCTGAATTAGTTCAATCATTTGTTGAACGACGTAAAGGTA
AAGCGACTGAAGAACAAGCACAAGAATTATTAAACAATGTGAACTACTTCGGTACAAT
GCTTGTTTATGCTGGTAAAGCAGATGGCTTAGTTAGTGGTGCAGCACATTCAACAGGCG
ACACTGTGCGTCCAGCATTACAAATCATCAAAACGAAACCAGGTGTATCAAGAACATC
AGGTATCTTCTTTATGATTAAAGGTGATGAACAATACATCTTTGGTGATTGTGCAATCAA
TCCAGAACTTGATTCACAAGGACTTGCAGAAATTGCAGTAGAAAGTGCAAAATCAGCA
TTA
>tpi_26
CACGAAACAGATGAAGAAATTAACAAAAAAGCGCATGCTATTTTCAAACATGGTATGA
CACCAATTATTTGTGTTGGTGAAACAGATGAAGAGCGTGAAAGTGGTAAAGCTAACGA
TGTTGTAGGTGAGCAAGTTAAGAAAGCTGTTGCAGGTTTATCTGAAGAGCAACTTAAA
TCAGTTGTAATTGCTTATGAACCAATCTGGGCAATCGGAACTGGTAAATCATCAACATC
TGAAGATGCGAATGAAATGTGTGCATTTGTACGTCAAACTATTGCTGACTTATCAAGCA
AAGAAGTATCAGAAGCAACTCGTATTCAATATGGTGGTAGTGTTAAACCTAACAACATT
AAAGAATACATGGCACAAACTGATATTGATGGGGCATTAGTAGGTGGCGCA
>yqil39

GCGTTTAAAGACGTGCCAGCCTATGATTTAGGTGCGACTTTAATAGAACATATTATTAAA
GAGACGGGTTTGAATCCAAGTGAGATTAATGAAGTCATCATCGGTAACGTACTACAAG
CAGGACAAGGACAAAATCCAGCACGAATTGCTGCTATGAAAGGTGGCTTGCCAGAAA
CAGTACCTGCATTTACAGTGAATAAAGTATGTGGTTCTGGGTTAAAGTCGATTCAATTA
GCATATCAATCTATTGTGACTGGTGAAAATGACATCGTGCTAGCTGGCGGTATGGAGAA
TATGTCTCAATCACCAATGCTTGTCAACAACAGTCGCTTTGGTTTTAAAATGGGACATC
AGTCAATGGTTGATAGCATGGTATATGATGGTTTAACAGATGTATTTAATCAATATCATAT
GGGTATTACTGCTGAAAATTTAGTAGAGCAATATGGTATTTCAAGAGAAGAACAAGATA
CATTTGCTGTAAACTCACAACAAAAAGCAGTACGTGCACAGCAA

ST1777

>arcc139
TTATTAATCCAACAAGCTAAATCGAACAGTGACACAACGCCGGCAATGCCATTGGATAC
TTGTGGTGCAATGTCACAAGGTATGATAGGCTATTGGTTGGAAACTGAAATCAATCGCA
TTTTAACTGAAATGAATAGTGATAGAACTGTAGGCACAATCGTAACACGTGTGGAAGTA
GATAAAGATGATCCACGATTTGATAACCCAACTAAACCAATTGGTCCTTTTTATACGAA
AGAAGAAGTTGAAGAATTACAAAAAGAACAGCCAGGCTCAGTCTTTAAAGAAGATGC
AGGACGTGGTTATAGAAAAGTAGTTGCGTCACCACTACCTCAATCTATACTAGAACATC
AGTTAATTCGAACTTTAGCAGACGGTAAAAATATTGTCATTGCATGCGGTGGTGGCGGT
ATTCCAGTTATAAAAAAAGAAAATACCTATGAAGGTGTTGAAGCG
>aroe2
AATTTTAATTCTTTAGGATTAGATGATACTTATGAAGCTTTAAATATTCCAATTGAAGATT
TTCATTTAATTAAAGAAATTATTTCAAAAAAAGAATTAGATGGCTTTAATATCACAATTC
CTCATAAAGAGCGTATCATACCGTATTTAGATCATGTTGATGAACAAGCGATTAATGCAG
GTGCAGTTAACACTGTTTTGATAAAAGATGGCAAGTGGATAGGGTATAATACAGATGGT
ATTGGTTATGTTAAAGGATTGCACAGCGTTTATCCAGATTTAGAAAATGCATACATTTTA
ATTTTGGGAGCAGGTGGTGCAAGTAAAGGTATTGCTTATGAATTAGCAAAATTTGTAAA
GCCCAAATTAACTGTTGCGAATAGAACGATGGCTCGTTTTGAATCTTGGAATTTAAATAT
AAACCAAATTTCATTGGCAGATGCTGAAAAGTATTTA
>glpf2
GGTGCTGATTGGATTGTCATCACAGCTGGATGGGGATTAGCGGTTACAATGGGTGTGTA
TGCTGTTGGTCAATTCTCAGGTGCACATTTAAACCCAGCGGTGTCTTTAGCTCTTGCAT
TAGACGGAAGTTTTGATTGGTCATTAGTTCCTGGTTATATTGTTGCTCAAATGTTAGGTG
CAATTGTCGGAGCAACAATTGTATGGTTAATGTACTTGCCACATTGGAAAGCGACAGA
AGAAGCTGGCGCGAAATTAGGTGTTTTCTCTACAGCACCGGCTATTAAGAATTACTTTG
CCAACTTTTTAAGTGAAATTATCGGAACAATGGCATTAACTTTAGGTATTTTATTTATCG
GTGTAAACAAAATTGCTGATGGTTTAAATCCTTTAATTGTCGGAGCATTAATTGTTGCAA
TCGGATTAAGTTTAGGCGGTGCTACTGGTTATGCAATCAACCCAGCACGT
>gmk_2
CGAATATTTGAAGATCCAAGTACATCATATAAGTATTCTATTTCAATGACAACACGTCAA
ATGCGTGAAGGTGAAGTTGATGGCGTAGATTACTTTTTTAAAACTAGGGATGCGTTTGA
AGCTTTAATTAAAGATGACCAATTTATAGAATATGCTGAATATGTAGGCAACTATTATGG
TACACCAGTTCAATATGTTAAAGATACAATGGACGAAGGTCATGATGTATTTTTAGAAAT
TGAAGTAGAAGGTGCAAAGCAAGTTAGAAAGAAATTTCCAGATGCGTTATTTATTTTCT

TAGCACCTCCAAGTTTAGATCACTTGAGAGAGCGATTAGTAGGTAGAGGAACAGAATC
TGATGAGAAAATACAAAGTCGTATTAACGAAGCACGTAAAGAAGTCGAAATGATGAAT
TTA
>pta_6
GCAACACAATTACAAGCAACAGATTATGTTACACCAATCGTGTTAGGTGATGAGACTAA
GGTTCAATCTTTAGCGCAAAAACTTAATCTTGATATTTCTAATATTGAATTAATTAATCCT
GCGACAAGTGAATTGAAAGCTGAATTAGTTCAATCATTTGTTGAACGACGTAAAGGTA
AAGCGACTGAAGAACAAGCACAAGAATTATTAAACAATGTGAACTACTTCGGTACAAT
GCTTGTTTATGCTGGTAAAGCAGATGGTTTAGTTAGTGGTGCAGCACATTCAACAGGCG
ACACTGTGCGTCCAGCTTTACAAATCATCAAAACGAAACCAGGTGTATCAAGAACATC
AGGTATCTTCTTTATGATTAAAGGTGATGAACAGTACATCTTTGGTGATTGTGCAATCAA
TCCAGAACTTGATTCACAAGGACTTGCAGAAATTGCAGTAGAAAGTGCAAAATCAGCA
TTA
>tpi_3
CACGAAACAGATGAAGAAATTAACAAAAAAGCGCACGCTATTTTCAAACATGGAATGA
CTCCAATTATTTGTGTTGGTGAAACAGACGAAGAGCGTGAAAGTGGTAAAGCTAACGA
TGTTGTAGGTGAGCAAGTTAAGAAAGCTGTTGCAGGTTTATCTGAAGATCAACTTAAAT
CAGTTGTAATTGCTTATGAACCAATCTGGGCAATCGGAACTGGTAAATCATCAACATCT
GAAGATGCGAATGAAATGTGTGCATTTGTACGTCAAACTATTGCTGACTTATCAAGCAA
AGAAGTATCAGAAGCAACTCGTATTCAATATGGTGGTAGTGTTAAACCTAACAACATTA
AAGAATACATGGCACAAACTGATATTGATGGGGCATTAGTAGGTGGCGCA
>yqil2
GCGTTTAAAGACGTGCCAGCCTATGATTTAGGTGCGACTTTAATAGAACATATTATTAAA
GAGACGGGTTTGAATCCAAGTGAGATTAATGAAGTCATCATCGGTAACGTACTACAAG
CAGGACAAGGACAAAATCCAGCACGAATTGCTGCTATGAAAGGTGGCTTGCCAGAAA
CAGTACCTGCATTTACAGTGAATAAAGTATGTGGTTCTGGGTTAAAGTCGATTCAATTA
GCATATCAATCTATTGTGACTGGTGAAAATGACATCGTGCTAGCTGGCGGTATGGAGAA
TATGTCTCAATCACCAATGCTTGTCAACAACAGTCGCTTTGGTTTTAAAATGGGACATC
AATCAATGGTTGATAGCATGGTATATGATGGTTTAACAGATGTATTTAATCAATATCATAT
GGGTATTACTGCTGAAAATTTAGTAGAGCAATATGGTATTTCAAGAGAAGAACAAGATA
CATTTGCTGTAAACTCACAACAAAAAGCAGTACGTGCACAGCAA

ST30
>arcc2
TTATTAATCCAACAAGCTAAATCGAACAGTGACACAACGCCGGCAATGCCATTGGATAC
TTGTGGTGCAATGTCACAAGGTATGATAGGCTATTGGTTGGAAACTGAAATCAATCGCA
TTTTAACTGAAATGAATAGTGATAGAACTGTAGGCACAATCGTAACACGTGTGGAAGTA
GATAAAGATGATCCACGATTTGATAACCCAACTAAACCAATTGGTCCTTTTTATACGAA
AGAAGAAGTTGAAGAATTACAAAAAGAACAGCCAGGCTCAGTCTTTAAAGAAGATGC
AGGACGTGGTTATAGAAAAGTAGTTGCGTCACCACTACCTCAATCTATACTAGAACACC
AGTTAATTCGAACTTTAGCAGACGGTAAAAATATTGTCATTGCATGCGGTGGTGGCGGT
ATTCCAGTTATAAAAAAAGAAAATACCTATGAAGGTGTTGAAGCG
>aroe2
AATTTTAATTCTTTAGGATTAGATGATACTTATGAAGCTTTAAATATTCCAATTGAAGATT

TTCATTTAATTAAAGAAATTATTTCAAAAAAAGAATTAGATGGCTTTAATATCACAATTC
CTCATAAAGAGCGTATCATACCGTATTTAGATCATGTTGATGAACAAGCGATTAATGCAG
GTGCAGTTAACACTGTTTTGATAAAAGATGGCAAGTGGATAGGGTATAATACAGATGGT
ATTGGTTATGTTAAAGGATTGCACAGCGTTTATCCAGATTTAGAAAATGCATACATTTTA
ATTTTGGGAGCAGGTGGTGCAAGTAAAGGTATTGCTTATGAATTAGCAAAATTTGTAAA
GCCCAAATTAACTGTTGCGAATAGAACGATGGCTCGTTTTGAATCTTGGAATTTAAATAT
AAACCAAATTTCATTGGCAGATGCTGAAAAGTATTTA
>glpf2
GGTGCTGATTGGATTGTCATCACAGCTGGATGGGGATTAGCGGTTACAATGGGTGTGTA
TGCTGTTGGTCAATTCTCAGGTGCACATTTAAACCCAGCGGTGTCTTTAGCTCTTGCAT
TAGACGGAAGTTTTGATTGGTCATTAGTTCCTGGTTATATTGTTGCTCAAATGTTAGGTG
CAATTGTCGGAGCAACAATTGTATGGTTAATGTACTTGCCACATTGGAAAGCGACAGA
AGAAGCTGGCGCGAAATTAGGTGTTTTCTCTACAGCACCGGCTATTAAGAATTACTTTG
CCAACTTTTTAAGTGAAATTATCGGAACAATGGCATTAACTTTAGGTATTTTATTTATCG
GTGTAAACAAAATTGCTGATGGTTTAAATCCTTTAATTGTCGGAGCATTAATTGTTGCAA
TCGGATTAAGTTTAGGCGGTGCTACTGGTTATGCAATCAACCCAGCACGT
>gmk_2
CGAATATTTGAAGATCCAAGTACATCATATAAGTATTCTATTTCAATGACAACACGTCAA
ATGCGTGAAGGTGAAGTTGATGGCGTAGATTACTTTTTAAAACTAGGGATGCGTTTGA
AGCTTTAATTAAAGATGACCAATTTATAGAATATGCTGAATATGTAGGCAACTATTATGG
TACACCAGTTCAATATGTTAAAGATACAATGGACGAAGGTCATGATGTATTTTTAGAAAT
TGAAGTAGAAGGTGCAAAGCAAGTTAGAAAGAAATTTCCAGATGCGTTATTTATTTTCT
TAGCACCTCCAAGTTTAGATCACTTGAGAGAGCGATTAGTAGGTAGAGGAACAGAATC
TGATGAGAAAATACAAAGTCGTATTAACGAAGCACGTAAAGAAGTCGAAATGATGAAT
TTA
>pta_6
GCAACACAATTACAAGCAACAGATTATGTTACACCAATCGTGTTAGGTGATGAGACTAA
GGTTCAATCTTTAGCGCAAAAACTTAATCTTGATATTTCTAATATTGAATTAATTAATCCT
GCGACAAGTGAATTGAAAGCTGAATTAGTTCAATCATTTGTTGAACGACGTAAAGGTA
AAGCGACTGAAGAACAAGCACAAGAATTATTAAACAATGTGAACTACTTCGGTACAAT
GCTTGTTTATGCTGGTAAAGCAGATGGTTTAGTTAGTGGTGCAGCACATTCAACAGGCG
ACACTGTGCGTCCAGCTTTACAAATCATCAAAACGAAACCAGGTGTATCAAGAACATC
AGGTATCTTCTTTATGATTAAAGGTGATGAACAGTACATCTTTGGTGATTGTGCAATCAA
TCCAGAACTTGATTCACAAGGACTTGCAGAAATTGCAGTAGAAAGTGCAAAATCAGCA
TTA
>tpi_3
CACGAAACAGATGAAGAAATTAACAAAAAAGCGCACGCTATTTTCAAACATGGAATGA
CTCCAATTATTTGTGTTGGTGAAACAGACGAAGAGCGTGAAAGTGGTAAAGCTAACGA
TGTTGTAGGTGAGCAAGTTAAGAAAGCTGTTGCAGGTTTATCTGAAGATCAACTTAAAT
CAGTTGTAATTGCTTATGAACCAATCTGGGCAATCGGAACTGGTAAATCATCAACATCT
GAAGATGCGAATGAAATGTGTGCATTTGTACGTCAAACTATTGCTGACTTATCAAGCAA
AGAAGTATCAGAAGCAACTCGTATTCAATATGGTGGTAGTGTTAAACCTAACAACATTA
AAGAATACATGGCACAAACTGATATTGATGGGGCATTAGTAGGTGGCGCA
>yqil2

GCGTTTAAAGACGTGCCAGCCTATGATTTAGGTGCGACTTTAATAGAACATATTATTAAA
GAGACGGGTTTGAATCCAAGTGAGATTAATGAAGTCATCATCGGTAACGTACTACAAG
CAGGACAAGGACAAAATCCAGCACGAATTGCTGCTATGAAAGGTGGCTTGCCAGAAA
CAGTACCTGCATTTACAGTGAATAAAGTATGTGGTTCTGGGTTAAAGTCGATTCAATTA
GCATATCAATCTATTGTGACTGGTGAAAATGACATCGTGCTAGCTGGCGGTATGGAGAA
TATGTCTCAATCACCAATGCTTGTCAACAACAGTCGCTTTGGTTTTAAAATGGGACATC
AATCAATGGTTGATAGCATGGTATATGATGGTTTAACAGATGTATTTAATCAATATCATAT
GGGTATTACTGCTGAAAATTTAGTAGAGCAATATGGTATTTCAAGAGAAGAACAAGATA
CATTTGCTGTAAACTCACAACAAAAAGCAGTACGTGCACAGCAA

ST45
>arcc10
TTATTAATCCAACAAGCTAAATCGAACAGTGACACAACGCCGGCAATGCCATTGGATAC
TTGTGGTGCAATGTCACAGGGTATGATAGGCTATTGGTTGGAAACTGAAATCAATCGCA
TTTTAACTGAAATGAATAGTGATAGAACTGTAGGCACAATCGTAACACGTGTGGAAGTA
GATAAAGATGATCCACGATTCAATAACCCAACCAAACCAATTGGTCCTTTTTATACGAA
AGAAGAAGTTGAAGAATTACAAAAAGAACAGCCAGACTCAGTCTTTAAAGAAGATGC
AGGACGTGGTTATAGAAAAGTAGTTGCGTCACCACTACCTCAATCTATACTAGAACACC
AGTTAATTCGAACTTTAGCAGACGGTAAAAATATTGTCATTGCATGCGGTGGTGGCGGT
ATTCCAGTTATAAAAAAAGAAAATACCTATGAAGGTGTTGAAGCG
>aroe14
AATTTTAATTCTTTGGGATTAGATGATACTTATGAAGCTTTAAATATTCCAATTGAAGATT
TTCATTTAATTAAAGAAATTATTTCAAAAAAAGAATTAGATGGCTTTAATATCACAATTC
CTCATAAAGAGCGTATCATACCGTATTTAGATCATGTTGATGAACAAGCGATTAATGCAG
GTGCAGTTAATACTGTTTTGATAAAAGATGGCAAGTGGATAGGGTATAATACAGATGGT
ATTGGTTATGTAAAAGGATTGCACAGCGTTTATCCAGATTTAGAAAATGCATACATTTTA
ATTTTGGGAGCAGGTGGTGCAAGTAAAGGTATTGCTTATGAATTAGCAAAATTTGTAAA
GCCCAAATTAACTGTTGCGAATAGAACGATGGCTCGTTTTGAATCTTGGAATTTAAATAT
AAACCAAATTTCATTGGCAGATGCTGAAAAGTATTTA
>glpf8
GGTGCTGATTGGATTGTCATCACAGCTGGATGGGGATTAGCGGTTACAATGGGTGTATA
TGCTGTCGGTCAATTCTCAGGTGCACATTTAAACCCAGCGGTGTCTTTAGCTCTTGCAT
TAGACGGAAGTTTTGATTGGTCATTAGTTCCTGGTTATATTGTTGCTCAAATGTTAGGTG
CAATTGTCGGAGCAACGATTGTATGGTTAATGTACTTGCCACATTGGAAAGCGACAGA
AGAAGCTGGCGCGAAATTAGGTGTTTTCTCTACAGCACCGGCTATTAAGAATTACTTTG
CCAACTTTTTAAGTGAGATTATCGGAACAATGGCATTAACTTTAGGTATTTTATTTATCG
GTGTAAACAAAATTGCCGATGGTTTAAATCCTTTAATTGTCGGAGCATTAATTGTTGCA
ATTGGATTAAGTTTAGGCGGTGCTACTGGTTATGCAATCAACCCAGCACGT
>gmk_6
CGAATATTTGAAGATCCAAGTACATCATATAAGTATTCTATTTCAATGACAACACGTCAA
ATGCGTGAAGGTGAAGTTGATGGCGTAGATTACTTTTTTAAAACTAGGGATGCGTTTGA
AGCTTTAATTAAAGATGACCAATTTATAGAATATGCTGAATATGTAGGCAACTATTATGG
TACACCAGTTCAATATGTTAAAGATACAATGGACGAAGGTCATGATGTATTTTTAGAAAT
TGAAGTAGAAGGTGCAAAGCAAGTTAGAAAGAAATTTCCAGATGCGTTATTTATTTTCT

TAGCACCTCCAAGTTTAGATCACTTGAGAGAGCGATTAGTAGGTAGAGGAACAGAATC
TGATGAGAAAATACAAAGTCGTATTAACGAAGCACGTAAAGAAGTTGAAATGATGAAT
TTA

>pta_10

GCAACACAATTACAAGCAACAGATTATGTTACACCAATCGTGTTAGGTGATGAGACTAA
GGTTCAATCTTTAGCGCAAAAACTTAATCTTGATATTTCTAATATTGAATTAATTAATCCT
GCGACAAGTGAATTGAAAGCTGAATTAGTTCAATCATTTGTTGAACGACGTAAAGGTA
AAGCGACTGAAGAACAAGCACAAGAATTATTAAACAATGTGAACTACTTCGGTACAAT
GCTTGTTTATGCTGGTAAAGCAGATGGTTTAGTTAGTGGTGCAGCACATTCAACAGGCG
ACACTGTGCGTCCAGCATTACAAATCATCAAAACGAAACCAGGTGTATCAAGAACATC
AGGTATCTTCTTTATGATTAAAGGTGATGAACAATACATCTTTGGTGATTGTGCAATCAA
TCCAGAACTTGATTCACAAGGACTTGCAGAAATTGCAGTAGAAAGTGCAAAATCAGCA
TTA

>tpi_3

CACGAAACAGATGAAGAAATTAACAAAAAAGCGCACGCTATTTTCAAACATGGAATGA
CTCCAATTATTTGTGTTGGTGAAACAGACGAAGAGCGTGAAAGTGGTAAAGCTAACGA
TGTTGTAGGTGAGCAAGTTAAGAAAGCTGTTGCAGGTTTATCTGAAGATCAACTTAAAT
CAGTTGTAATTGCTTATGAACCAATCTGGGCAATCGGAACTGGTAAATCATCAACATCT
GAAGATGCGAATGAAATGTGTGCATTTGTACGTCAAACTATTGCTGACTTATCAAGCAA
AGAAGTATCAGAAGCAACTCGTATTCAATATGGTGGTAGTGTTAAACCTAACAACATTA
AAGAATACATGGCACAAACTGATATTGATGGGGCATTAGTAGGTGGCGCA

>yqil2

GCGTTTAAAGACGTGCCAGCCTATGATTTAGGTGCGACTTTAATAGAACATATTATTAAA
GAGACGGGTTTGAATCCAAGTGAGATTAATGAAGTCATCATCGGTAACGTACTACAAG
CAGGACAAGGACAAAATCCAGCACGAATTGCTGCTATGAAAGGTGGCTTGCCAGAAA
CAGTACCTGCATTTACAGTGAATAAAGTATGTGGTTCTGGGTTAAAGTCGATTCAATTA
GCATATCAATCTATTGTGACTGGTGAAAATGACATCGTGCTAGCTGGCGGTATGGAGAA
TATGTCTCAATCACCAATGCTTGTCAACAACAGTCGCTTTGGTTTTAAAATGGGACATC
AATCAATGGTTGATAGCATGGTATATGATGGTTTAACAGATGTATTTAATCAATATCATAT
GGGTATTACTGCTGAAAATTTAGTAGAGCAATATGGTATTTCAAGAGAAGAACAAGATA
CATTTGCTGTAAACTCACAACAAAAAGCAGTACGTGCACAGCAA

ST7

>arcc5

TTATTAATCCAACAAGCTAAATCGAACAGTGACACAACGCCGGCAATGCCATTGGATAC
TTGTGGTGCAATGTCACAGGGTATGATAGGCTATTGGTTGGAAACTGAAATCAATCGCA
TTTTAACTGAAATGAATAGTGATAGAACTGTAGGCACAATCGTTACACGTGTGGAAGTA
GATAAAGATGATCCACGATTTGATAACCCAACTAAACCAATTGGTCCTTTTTATACGAA
AGAAGAAGTTGAAGAATTACAAAAAGAACAGCCAGACTCAGTCTTTAAAGAAGATGC
AGGACTTGGTTATAGAAAAGTAGTTGCGTCACCACTACCTCAATCTATACTAGAACACC
AGTTAATTCGAACTTTAGCAGACGGTAAAAATATTGTCATTGCATGCGGTGGTGGCGGT
ATTCCAGTTATAAAAAAAGAAAATACCTATGAAGGTGTTGAAGCG

>aroe4

AATTTTAATTCTTTAGGATTAGATGATACTTATGAAGCTTTAAATATTCCAATTGAAGATT

TTCATTTAATTAAAGAAATTATTTCGAAAAAAGAATTAGATGGCTTTAATATCACAATTC
CTCATAAAGAACGTATCATACCGTATTTAGATTATGTTGATGAACAAGCGATTAATGCAG
GTGCAGTTAACACTGTTTTGATAAAAGATGGCAAGTGGATAGGGTATAATACAGATGGT
ATTGGTTATGTTAAAGGATTGCACAGCGTTTATCCAGATTTAGAAAATGCATACATTTTA
ATTTTGGGCGCAGGTGGTGCAAGTAAAGGTATTGCTTATGAATTAGCAAAATTTGTAAA
GCCCAAATTAACTGTTGCGAATAGAACGATGGCTCGTTTTGAATCTTGGAATTTAAATAT
AAACCAAATTTCATTGGCAGATGCTGAAAAGTATTTA
>glpf1
GGTGCTGATTGGATTGTCATCACAGCTGGATGGGGATTAGCGGTTACAATGGGTGTGTT
TGCTGTCGGTCAATTCTCAGGTGCACATTTAAACCCAGCGGTGTCTTTAGCTCTTGCAT
TAGACGGAAGTTTTGATTGGTCATTAGTTCCTGGTTATATTGTTGCTCAAATGTTAGGTG
CAATTGTCGGAGCAACAATTGTATGGTTAATGTACTTGCCACATTGGAAAGCGACAGA
AGAAGCTGGCGCGAAATTAGGTGTTTTCTCTACAGCACCGGCTATTAAGAATTACTTTG
CCAACTTTTTAAGTGAGATTATCGGAACAATGGCATTAACTTTAGGTATTTTATTTATCG
GTGTAAACAAAATTGCCGATGGTTTAAATCCTTTAATTGTCGGAGCATTAATTGTTGCA
ATCGGATTAAGTTTAGGCGGTGCTACTGGTTATGCAATCAACCCAGCACGT
>gmk_4
CGAATATTTGAAGATCCAAGTACATCATATAAGTATTCTATTTCAATGACAACACGTCAA
ATGCGTGAAGGTGAAGTTGATGGCGTAGATTACTTTTTAAAACTAGGGATGCGTTTGA
AGCTTTAATTAAAGATGACCAATTTATAGAATATGCTGAATATGTAGGCAACTATTATGG
TACACCAGTTCAATATGTTAAAGATACAATGGACGAAGGTCATGATGTATTTTTAGAAAT
TGAAGTAGAAGGTGCAAAGCAAGTTAGAAAGAAATTTCCAGATGCGTTATTTATTTTCT
TAGCACCTCCAAGTTTAGATCACTTGAGAGAGCGATTAGTAGGTAGAGGAACAGAATC
CAATGAGAAAATACAAAGTCGTATTAACGAAGCGCGTAAAGAAGTTGAAATGATGAAT
TTA
>pta_4
GCAACACAATTACAAGCAACAGATTATGTTACACCAATCGTGTTAGGTGATGAGACTAA
GGTTCAATCTTTAGCGCAAAAACTTGATCTTGATATTTCTAATATTGAATTAATTAATCCT
GCGACAAGTGAATTGAAAGCTGAATTAGTTCAATCATTTGTTGAACGACGTAAAGGTA
AAGCGACTGAAGAACAAGCACAAGAATTATTAAACAATGTGAACTACTTCGGTACAAT
GCTTGTTTATGCTGGTAAAGCAGATGGTTTAGTTAGTGGTGCAGCACATTCAACAGGCG
ACACTGTGCGTCCAGCTTTACAAATCATCAAAACGAAACCAGGTGTATCAAGAACATC
AGGTATCTTCTTTATGATTAAAGGTGATGAACAATACATCTTTGGTGATTGTGCAATCAA
TCCAGAACTTGATTCACAAGGACTTGCAGAAATTGCAGTAGAAAGTGCAAAATCAGCA
TTA
>tpi_6
CACGAAACAGATGAAGAAATTAACAAAAAAGCGCACGCTATTTTCAAACATGGAATGA
CTCCAATTATATGTGTTGGTGAAACAGACGAAGAGCGTGAAAGTGGTAAAGCTAACGA
TGTTGTAGGTGAGCAAGTTAAGAAAGCTGTTGCAGGTTTATCTGTAGATCAACTTAAAT
CAGTTGTAATTGCTTATGAACCAATCTGGGCAATCGGAACTGGTAAATCATCAACATCT
GAAGATGCAAATGAAATGTGTGCATTTGTACGTCAAACTATTGCTGACTTATCAAGCAA
AGAAGTATCAGAAGCAACTCGTATTCAATATGGTGGTAGTGTTAAACCTAACAACATTA
AAGAATACATGGCACAAACTGATATTGATGGGGCATTAGTAGGTGGCGCA
>yqil3

GCGTTTAAAGACGTGCCAGCCTATGATTTAGGTGCGACTTTAATAGAACATATTATTAAA
GAGACGGGTTTGAATCCAAGTGAGATTGATGAAGTTATCATCGGTAACGTACTACAAG
CAGGACAAGGACAAAATCCAGCACGAATTGCTGCTATGAAAGGTGGCTTGCCAGAAA
CAGTACCTGCATTTACGGTGAATAAAGTATGTGGTTCTGGGTTAAAGTCGATTCAATTA
GCATATCAATCTATTGTGACTGGTGAAAATGACATCGTGCTAGCTGGCGGTATGGAGAA
TATGTCTCAATCACCAATGCTTGTCAACAACAGTCGCTTTGGTTTTAAAATGGGACATC
AATCAATGGTTGATAGCATGGTATATGATGGTTTAACAGATGTATTTAATCAATATCATAT
GGGTATTACTGCTGAAAATTTAGTAGAGCAATATGGTATTTCAAGAGAAGAACAAGATA
CATTTGCTGTAAACTCACAACAAAAAGCAGTACGTGCACAGCAA
