# Supplementary material for: Comparison of community- and healthcare-associated methicillin-resistant Staphylococcus aureus isolates at a Chinese tertiary hospital, 2012–2017
Source: Sci Rep. 2018 Dec 17;8:17916. doi: 10.1038/s41598-018-36206-5 (PMC6297250; doi:10.1038/s41598-018-36206-5)
Supplement: Supplementary file 2 — Supplementary Table S2 [file 41598_2018_36206_MOESM2_ESM.pdf]

Comparison of community- and healthcare-associated methicillin-resistant *Staphylococcus aureus* isolates at a Chinese tertiary hospital, 2012–2017

Haiying Peng [1], Dengtao Liu [1], Yuhua Ma [1] & Wei Gao [1,*]

[1] Department of Clinical Laboratory, Linyi People's Hospital, Linyi, Shandong, China

Haiying Peng: phy1977@126.com

Dengtao Liu: 15969917998@163.com

Yuhua Ma: ma_yuhua@163.com

Wei Gao: gaoweisdly@126.com

***Corresponding author's mailing address:** Department of Clinical Laboratory, Linyi People's Hospital, 27 Jiefang Rd., Linyi, Shandong, People's Republic of China; Zip Code: 276003. Phone: +86-539-8219735. E-mail: gaoweisdly@126.com

Supplementary Table S2. Primers used for molecular typing.

Primers	Primer sequence (5'−3')	Product size (bp)
**For *spa* typing**		
spa-1095 F	AGACGATCCTTCGGTGAGC	variable size
spa-1517 R	GCTTTTGCAATGTCATTTACTG	
**For SCC*mec* typing**		
SCCmec I-F	GCTTTAAAGAGTGTCGTTACAGG	613
SCC*mec* I-R	GTTCTCTCATAGTATGACGTCC	
SCC*mec* II-F	CGTTGAAGATGATGAAGCG	398
SCC*mec* II-R	CGAAATCAATGGTTAATGGACC	
SCC*mec* III-F	CCATATTGTGTACGATGCG	280
SCC*mec* III-R	CCTTAGTTGTCGTAACAGATCG	
SCC*mec* IVa-F	GCCTTATTCGAAGAAACCG	776
SCC*mec* IVa-R	CTACTCTTCTGAAAAGCGTCG	
SCC*mec* IVb-F	TCTGGAATTACTTCAGCTGC	493
SCC*mec* IVb-R	AAACAATATTGCTCTCCCTC	
SCC*mec* IVc-F	ACAATATTTGTATTATCGGAGAGC	200
SCC*mec* IVc-R	TTGGTATGAGGTATTGCTGG	
SCC*mec* IVd-F	CTCAAAATACGGACCCCAATACA	881
SCC*mec* IVd-R	TGCTCCAGTAATTGCTAAAG	
SCC*mec* V-F	GAACATTGTTACTTAAATGAGCG	325
SCC*mec* V-R	TGAAAGTTGTACCCTTGACACC	
**For MLST typing**		
arcC-F	TTGATTCACCAGCGCGTATTGTC	456
arcC-R	AGGTATCTGCTTCAATCAGCG	
aroE-F	ATCGGAAATCCTATTTCACATTC	456
aroE-R	GGTGTTGTATTAATAACGATATC	
glpF-F	CTAGGAACTGCAATCTTAATCC	465
glpF-R	TGGTAAAATCGCATGTCCAATTC	
gmk-F	ATCGTTTTATCGGGACCATC	429
gmk-R	TCATTAACTACAACGTAATCGTA	
pta-F	GTTAAAATCGTATTACCTGAAGG	474
pta-R	GACCCTTTTGTTGAAAAGCTTAA	
tpi-F	TCGTTCATTCTGAACGTCGTGAA	402
tpi-R	TTTGCACCTTCTAACAATTGTAC	
yqiL-F	CAGCATACAGGACACCTATTGGC	516
yqiL-R	CGTTGAGGAATCGATACTGGAAC	
